# Evaluating the real-life effect of MP-AzeFlu on asthma outcomes in patients with allergic rhinitis and asthma in UK primary care

**DOI:** 10.1016/j.waojou.2020.100490

**Published:** 2020-12-19

**Authors:** Hilda J.I. De Jong, Jaco Voorham, Glenis K. Scadding, Claus Bachert, Giorgio Walter Canonica, Peter Smith, Ulrich Wahn, Dermot Ryan, Jose A. Castillo, Victoria A. Carter, Ruth B. Murray, David B. Price

**Affiliations:** a Observational and Pragmatic Research Institute, Singapore; b Royal National Throat, Nose and Ear Hospital, University College London School of Medicine, London, UK; c Ghent University Hospital, Ghent, Belgium; d Personalized Medicine Asthma & Allergy Clinic, Humanitas University & Research Hospital, SANI-Severe Asthma Network, Milan, Italy; e Griffith University, Southport, QLD, Australia; f Charité Medical University, Berlin, Germany; g Usher Institute, University of Edinburgh, Edinburgh, UK; h Optimum Patient Care, Cambridge, UK; i Hospital Universitari Quirón Dexeus, Barcelona, Spain; j Academic Primary Care, University of Aberdeen, Aberdeen, UK

**Keywords:** Control, Exacerbations, Rescue medication, ADEPT, Anonymized data ethics & protocol transparency, AR, Allergic rhinitis, ATS, American Thoracic society, BEC, Blood eosinophil count, CRS, Chronic rhinosinusitis, ERS, European respiratory society, FEV_1_, forced expiratory volume in 1 s, FVC, Forced vital capacity, GERD, Gastroesophageal reflux disease, GINA, Global initiative for asthma, ICS, Inhaled corticosteroid, INS, Intranasal corticosteroid, NP, Nasal polyps, OAC, Overall asthma control, OAH, Oral anti-histamine, OCS, Oral corticosteroid, OPCRD, Optimum patient care research database, OTC, Over the counter, PEF, Peak expiratory flow rate, RCT, Randomized controlled trial, RDAC, Risk domain asthma control, SABA, Short-acting β_2_-agonist, SMD, Standardised mean difference, UK, United Kingdom

## Abstract

**Background:**

MP-AzeFlu (Dymista®; spray of azelastine/fluticasone propionate) is the most effective allergic rhinitis (AR) treatment available. Its effect on asthma outcomes in patients with AR and asthma is unknown.

**Methods:**

This pre-post historical cohort study, using the Optimum Patient Care Research Database, included patients aged ≥12 years, from UK general practice with active asthma (defined as a recorded diagnosis, with ≥1 prescription for reliever or controller inhaler) in the year before or at the initiation date. The primary study outcome was change in number of acute respiratory events (i.e. exacerbation or antibiotic course for a respiratory event) between baseline and outcome years. The effect size of MP-AzeFlu was quantified as the difference in % of patients that improved and worsened.

**Results:**

Of the 1,188 patients with AR and asthma included, many had a record of irreversible obstruction (67%), and uncontrolled asthma (70.4%), despite high mean daily doses of reliever/controller therapy and acute oral corticosteroid use, in the year pre-MP-AzeFlu initiation. MP-AzeFlu initiation was associated with fewer acute respiratory events (effect size (e) = 5.8%, p = 0.0129) and a reduction in daily use of short-acting β_2_-agonists, with fewer patients requiring >2 SABA puffs/week (e = 7.7% p < 0.0001). More patients had well-controlled asthma 1-year post-MP-AzeFlu initiation (e = 4.1%; p = 0.0037), despite a reduction in inhaled corticosteroids (e = 4.8%; p = 0.0078).

**Conclusions:**

This study provides the first direct evidence of the beneficial effect of MP-AzeFlu on asthma outcomes in co-morbid patients in primary care in the United Kingdom.

**Trial registration:**

EUPAS30940. Registered August 13, 2019.

## Introduction

Allergic diseases are complex and can cluster in multi-morbidities.^1^ One such example is the concurrence of allergic asthma and rhinitis in the same patient.^2^, ^3^, ^4^ These diseases are linked on several levels, often referred to as the “one airway one disease” or “unified airway disease” concept.^5^^,^^6^ Evidence to support this link comes from epidemiological, pathophysiological, clinical, and socioeconomic studies.^2^^,^^3^ For example, the prevalence of asthma is more than 6 times higher in those with rhinitis than in those without.^7^ Both diseases share contiguous anatomy, triggers, and inflammatory processes.^2^^,^^3^^,^^8^ Control of these diseases is also linked, with control of one affecting control of the other.^9^ Furthermore, co=morbid patients experience more asthma exacerbations,^10^^,^^11^ use more asthma medication,^12^ and have more physician and hospital visits for their asthma.^13^^,^^14^

Taking this into account, reducing inflammation in the nose should improve outcomes in the lungs for those patients with both diseases, a theory endorsed by allergic rhinitis (AR) management guidelines.^8^^,^^15^ Treating both asthma and AR together results in better asthma outcomes, including an improvement in lung function, and a reduction in asthma-related hospitalizations and exacerbations.^16^, ^17^, ^18^ The “AR treatment efficacy hierarchy concept” takes this one step further, postulating that more effective AR control should, in turn, have a greater positive impact on multi-morbid asthma control. Part of this hierarchy has already been proven: intranasal corticosteroids (INS) provide better AR symptom control than oral anti-histamines (OAHs),^19^ and as a consequence, unlike OAHs, INS have a positive impact on many asthma outcomes, including lung function and reduced asthma rescue medication use.^20^^,^^21^ However, many AR patients remain symptomatic on INS monotherapy (and multiple therapies),^22^ perhaps due to a recently identified INS efficacy ceiling.^23^ Could a more effective AR treatment confer greater synergistic benefits for asthma outcomes in co-morbid patients?

MP-AzeFlu comprises an INS (fluticasone propionate; FP) and an intranasal anti-histamine (azelastine; AZE) in a patented formulation, delivered in a single spray. In the United Kingdom, MP-AzeFlu has been available since 2013 on prescription only, and it is fully reimbursed, subject to a standardised co-pay that applies to all UK prescriptions. It is indicated for the treatment of both seasonal and perennial AR.^24^ It is currently the most effective symptomatic pharmacotherapy for the treatment of AR (twice as effective as an INS), with the fastest onset of action (5 min).^23^^,^^25^, ^26^, ^27^ Although, socioeconomic evidence for MP-AzeFlu's positive effect on asthma control in AR and asthma co=morbid patients has previously been published,^28^ a direct effect on asthma control is missing. An assessment of the effect of AR treatment on asthma outcomes in real-life is also warranted; previous evidence with INS has come from randomized controlled trials (RCT_s_),^21^ the results of which may not be generalizable to the wider AR and asthma population seen in routine clinical care.^29^, ^30^, ^31^ The aim of the current study was to investigate the real-life effect of MP-AzeFlu on asthma outcomes in patients with both AR and asthma in UK primary care.

## Methods

### Study design

**Fig. 1:**
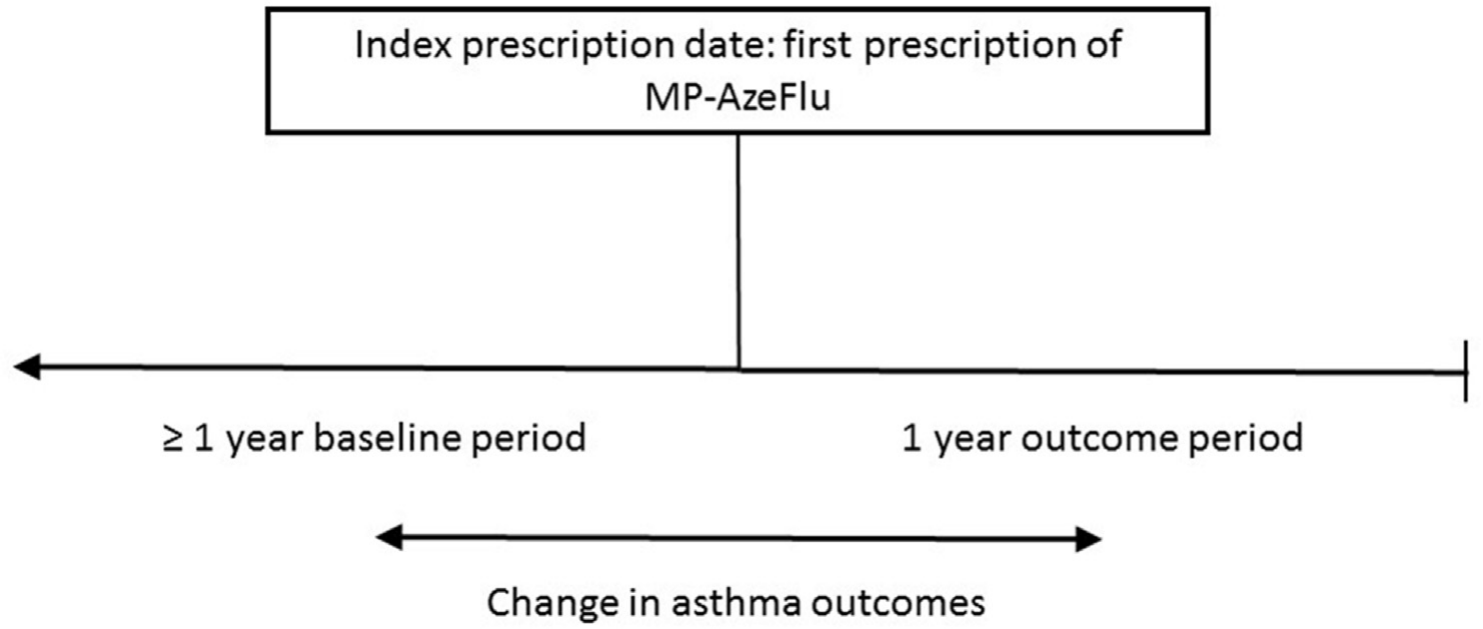
Pre-post, historical, cohort study design.

This was a 2-year, pre-post, historical cohort study, using data from the Optimum Patient Care Research Database (OPCRD),^32^ to compare asthma-related outcomes in the period before and after initiation of MP-AzeFlu (ie, index prescription date; Fig. 1). This self-controlled study design ensured that patient characteristics which were stable over the study period could not affect the associations of interest. The index prescription date (IPD) was the date at which patients received their first prescription of MP-AzeFlu. The OPCRD dataset contains patient records from June 1930 to March 2019; MP-AzeFlu first became available in the United Kingdom in 2013. Baseline data were captured at least a minimum of one year prior to MP-AzeFlu prescription, and outcome data (ie,. to assess the impact of MP-AzeFlu on asthma-related outcomes) were captured in the year after MP-AzeFlu prescription. The study protocol was approved by the Anonymized Data Ethics and Protocols Transparency (ADEPT) committee. ADEPT is an independent body of experts and regulators commissioned by the Respiratory Effectiveness Group to govern the standard of research conducted on internationally recognised databases.^33^ The study protocol was registered with the European Union electronic Register of Post-Authorization studies.^34^

### Data source

The OPCRD comprises medical records of more than 7 million patients from over 700 general practices across the United Kingdom (approximately 8% of the total UK population) and integrates with all UK clinical systems (EMIS, TPP SystmOne, InPS Vision, Microtest Evolution). It benefits from a long retrospective period (median time in the database is 13 years, goes back to birth for summary diagnostic data in many cases), and contains linked patient-completed respiratory questionnaires for approximately 10% of asthma patients included.^32^ Asthma-related outcome measures within the OPCRD have been validated using patient reported outcomes.^35^

### Patients

**Fig. 2:**
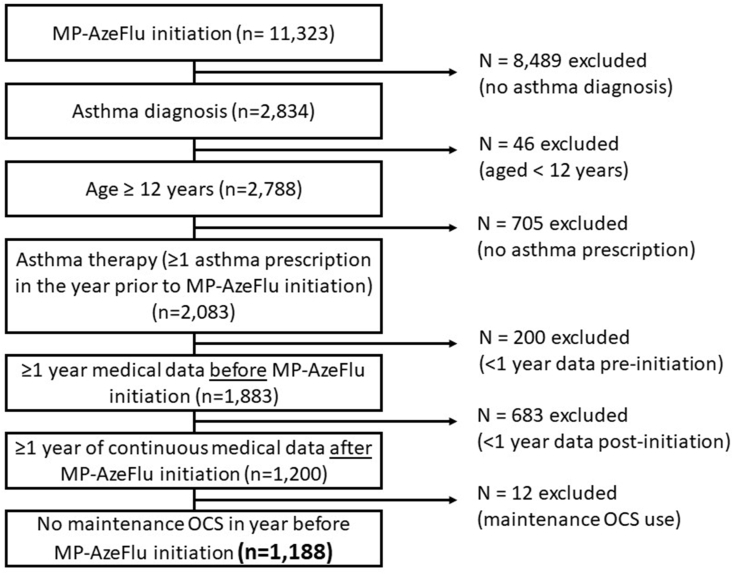
Subject disposition. Patients within the Optimum Patient Care Database who fulfilled study inclusion criteria. OCS: oral corticosteroids

Patients included in this analysis had received at least 1 prescription for MP-AzeFlu, were aged ≥ 12 years old (at IPD) and had “active” asthma (Fig. 2). As MP-AzeFlu is indicated solely for AR (both seasonal and perennial) in the United Kingdom, it was assumed that all patients included in the study had AR. Analysis of upper respiratory disease diagnostic codes ever, prior to MP-AzeFlu initiation showed marked coding overlap, reflecting patient journeys to and within medical care services in the United Kingdom and co-morbidity burdens ( S-Fig. 1). Active asthma was defined as ever having a recorded diagnostic Read code for asthma before starting MP-AzeFlu, and ≥1 asthma therapy prescription (ie, reliever and/or controller medication) in the year prior to MP-AzeFlu initiation. Eligible patients were required to have at least 2 years of data, comprising 1 year of data before (baseline year) and after MP-AzeFlu initiation (outcome year). Patients with only a diagnosis of chronic obstructive pulmonary disease ever before or a prescription for maintenance oral corticosteroids (OCS) and biologics in the year prior to MP-AzeFlu prescription were excluded (Fig. 2).

### Study outcomes

The primary outcome was change (from baseline year to outcome year) in the number of acute respiratory events. An acute respiratory event was defined when 1 or more of the following applied: an asthma-related primary care-recorded hospital admission; an asthma-related primary-care recorded accident and emergency(A&E) attendance; an acute course of OCS; an antibiotic course with evidence of respiratory consultation. This definition has previously been validated as part of the Risk Domain Asthma Control questionnaire.^35^ An occurrence of any of these factors within 14 days of each other was considered to belong to the same event (Table 1). The secondary outcome was change in the number of asthma exacerbations. An exacerbation was defined according to American Thoracic Society/European Respiratory Society (ATS/ERS). Task Force definition (Table 1).^36^ Exploratory outcomes included change in: (i) asthma Global Initiative for Asthma (GINA) 2018^37^ treatment step; (ii) asthma control; (iii) average daily dose of short-acting β_2_-agonist (SABA) or >2 puffs of SABA per week; and (iv) average daily dose of inhaled corticosteroids (ICS). Asthma control was assessed in 3 ways; risk domain asthma control (RDAC), overall asthma control (OAC) and GINA control status (ie, controlled, partly-controlled, and un-controlled).^35^^,^^37^^,^^38^ A definition of each of these outcomes is provided in Table 1 and in the online supplement under the section entitled "Study outcomes: definitions".

**Table 1:** Summary of asthma outcome definitions.

Term	Definition
Acute respiratory event	≥1 of any of: (i) asthma-related primary care-recorded hospital admission^a^, (ii) asthma-related primary care-recorded A&E attendance^b^, (iii) acute course of OCS, (iv) antibiotics course with evidence of respiratory consultation. Occurrences within 14 days of each other were considered to belong to the same event.
Asthma exacerbation	≥1 of any of: (i) asthma-related primary care-recorded hospital admission, (ii) asthma-related primary care-recorded A&E attendance, (iii) acute course of OCS. Occurrences within 14 days of each other were considered to belong to the same event.
GINA treatment step^37^	Highest step during the baseline and outcome years, with the daily dosage of ICS based on the last prescription in each period.
RDAC^38^	The absence of an acute respiratory event (see above) AND an asthma-related out-patient department (specialist) consultation in the baseline year.
OAC^38^	The absence of an acute respiratory event (see above) AND an asthma-related out-patient department (specialist) consultation AND an average daily dose of SABA >200 μg salbutamol/>500 μg terbutaline in the baseline year.
GINA control status^37^	Based on a yes/no response to GINA control question: • Day times symptoms >2/week • Any night wakening due to asthma • Reliever medication >2/week • Any activity limitation due to asthma 0 ‘yes’ = well controlled; 1–2 ‘yes’ = partly controlled; 3–4 ‘yes’ = uncontrolled
Average daily dose of SABA >2 puffs of SABA per week	Based on collected prescriptions in the baseline year. Calculated as count of inhalers × doses in pack × μg strength/365. Expressed as salbutamol equivalent in μg/day.>28,571 (more than 2 puffs a week) μg/day.
Average daily dose of ICS	Based on collected prescriptions in the baseline year. Calculated as count of inhalers × doses in pack × μg strength/365). Expressed as fluticasone propionate equivalent in μg/day.
Adherence	Calculated by the medication possession ratio. Refill rate (%) = (total ICS pack days/number of prescription days)∗100

A&E: Accident & Emergency; OCS: oral corticosteroids; ICS inhaled corticosteroid; RDAC: Risk Domain Asthma Control; OAC: overall asthma control; SABA: short-acting β_2_-agonist.

a Definite asthma hospital admission OR a generic hospitalization. Read code which has been recorded on the same day as a lower respiratory consultation.

b Definite asthma on emergency attendance OR a generic emergency. Hospital Read code which has been recorded on the same day as a lower respiratory consultation

### Statistical analysis

The Wilcoxon's signed rank test for paired data calculated that 1103 patients were required to detect a difference in the primary outcome variable (ie, number of acute respiratory events) between the outcome and baseline years, with an effect size of 0.1 and 90% statistical power at a significance level of 0.05. All outcome variables were defined a priori. Baseline variables with missing data were presented as the number of non-missing observations.

Summary statistics were used to describe the distribution of demographic and clinical characteristics in the year prior to IPD. The number of acute respiratory events, number of exacerbations, GINA treatment step, GINA control status, and average daily dose of SABA or ICS in the baseline and outcome years were compared using the Wilcoxon signed rank test (for paired data). Change in asthma control (assessed by RDAC and OAC) was assessed using the McNemar's test. A p-value of ≤0.05 was considered statistically significant. Each of these asthma outcomes was reported as the proportion of patients who improved, worsened and remained stable in the outcome year. The effect of the intervention was expressed as the difference in the percentage of patients improving and worsening on a certain asthma outcome.

These analyses were performed in the total population and for those: (i) with and without a prescription of INS in the past 45 days (ie, active vs not active INS use), (ii) ever or never treated with INS, (iii) who had 0 or 2 exacerbations in the year prior to the index date, and (iv) in those with blood eosinophil count (BEC) <0.25 and >0.25 10^9^/L in the 5 years prior to and up to MP-AzeFlu initiation, to assess whether these had an impact on asthma control variables assessed. In this self-controlled study design ICS may be a potential confounder (ie, the initiation of MP-AzeFlu may be associated with a change in ICS, and ICS may be associated with a change in asthma-related outcomes) and an analysis adjusting for ICS was also conducted (S-Table 2). In case of controlling for confounders, a conditional logistic regression was used for binary outcomes. Findings were reported as odds ratio (OR) and 95% CI. For count outcomes, a conditional negative binomial regression was used when we adjusted for confounders. The results were reported as rate ratio and corresponding 95% confidence interval (CI). For outcomes with more than two categories (eg, GINA step), adjustment for confounders conditional multinomial logistic regression was used. For this analysis the odds ratio (OR) and 95% CI were presented. All statistical analyses were performed using Stata MP6 V.15 and Stata SE V.14 (StataCorp, College Station, Texas, USA).

## Results

### Study population

From an initial 11323 patients in the OPCRD who received MP-AzeFlu treatment, 1188 patients met all inclusion criteria and were included in the analysis (Fig. 2).

### Baseline characteristics (year prior to MP-AzeFlu prescription)

#### Demographic

Patients who were prescribed MP-AzeFlu were most likely to be aged between 18 and 64 years old, female, over-weight/obese, with a smoking history (past or present; Table 2). The average (standard deviation) number of MP-AzeFlu prescriptions in the outcome year was 1.54 (2.74).

#### Baseline co-morbidities and rhinitis treatments

**Fig. 3:**
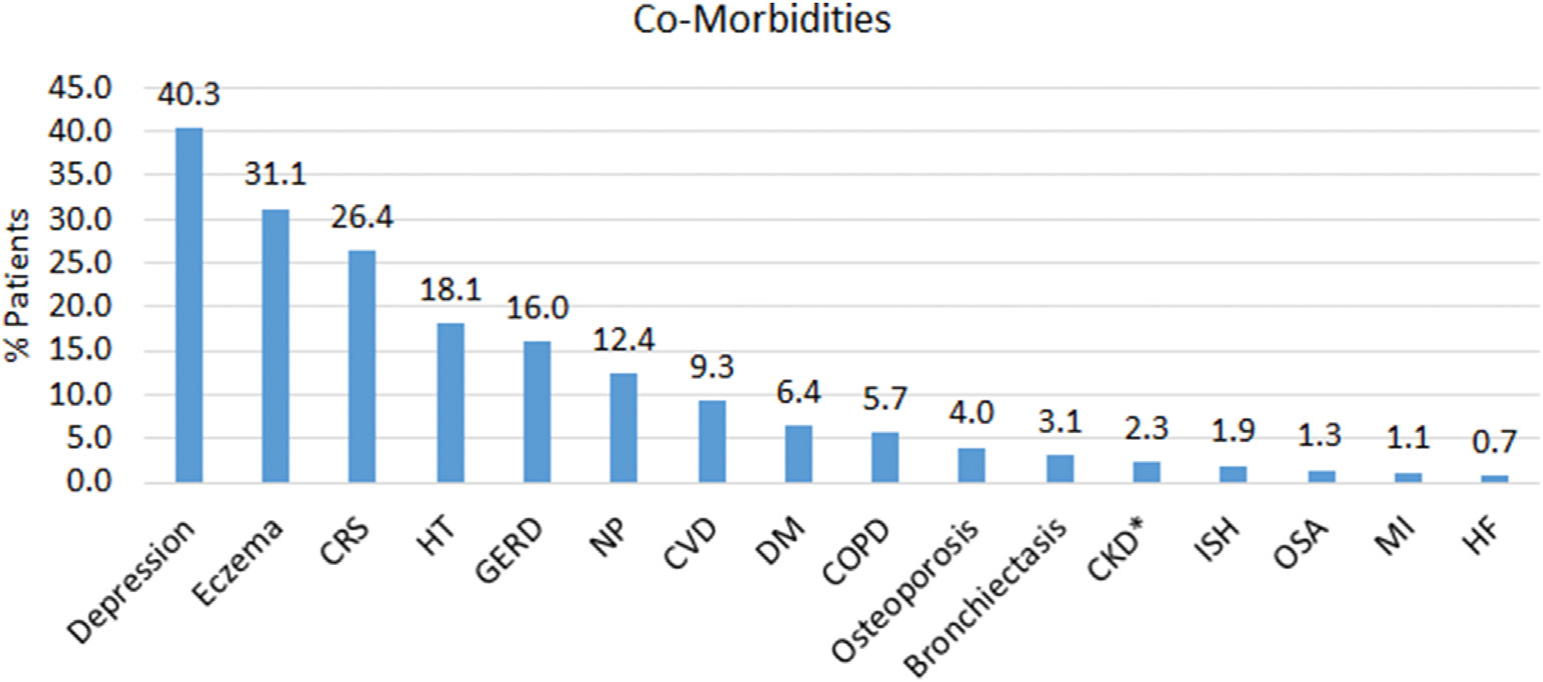
Co-morbidities experienced by patients with AR and active asthma (≥12 years old) attending primary care in the UK, in the year prior to MP-AzeFlu initiation (n = 1188).CRS: chronic rhinosinusitis; HT: hypertension; GERD: gastroesophageal reflex disease; NP: nasal polyps; CVD: cardiovascular disease; DM: diabetes mellitus; COPD: chronic obstructive pulmonary disease; CKD: chronic kidney disease (∗stage 3–5); ISH: Ischaemic heart disease; OSA: obstructive sleep apnoea; MI: myocardial infarction; HF: heart failure

**Fig. 4:**
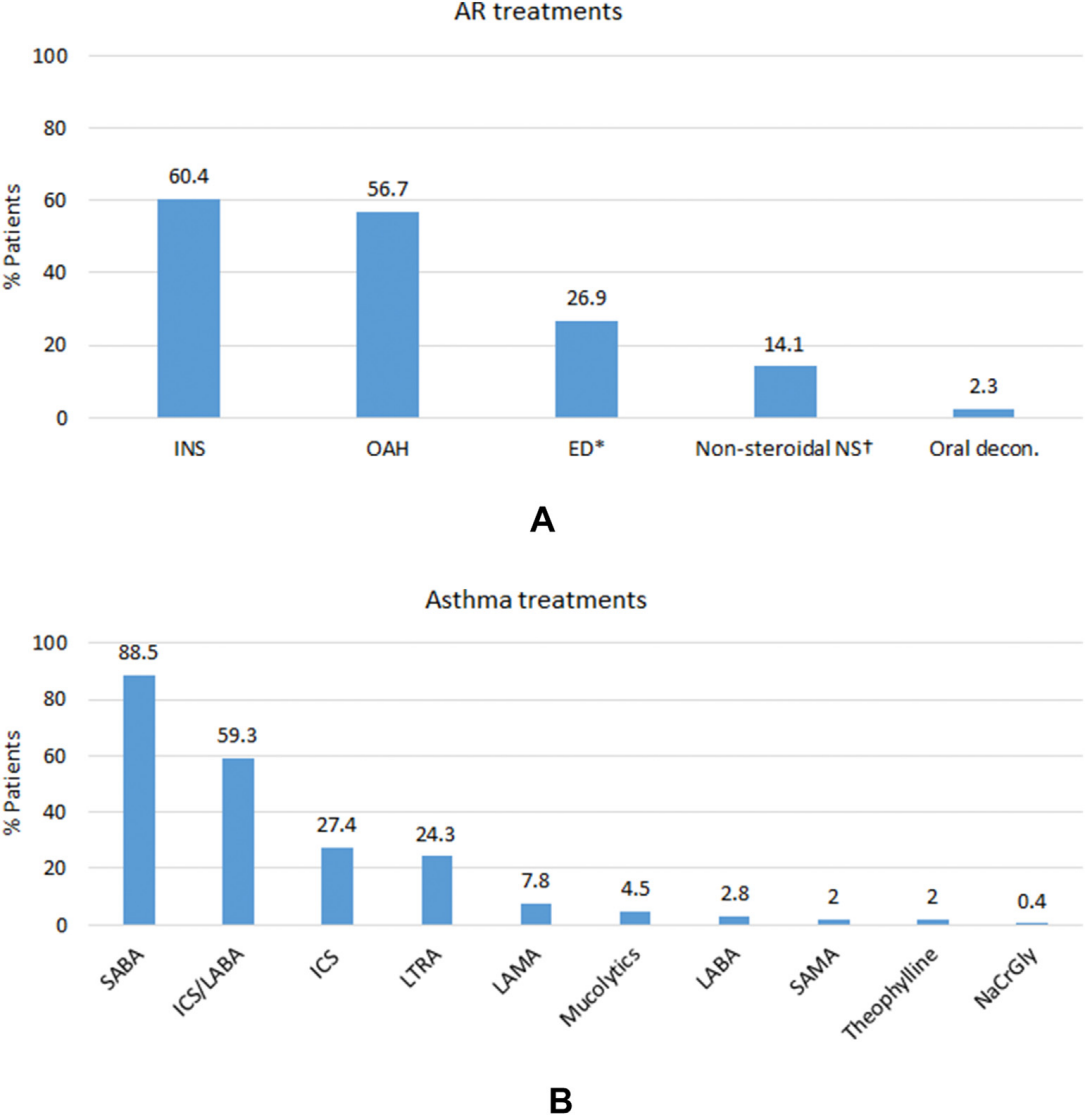
(A) allergic rhinitis (AR) treatments and (B) asthma treatments prescribed to patients with AR and active asthma (≥12 years old) attending primary care in the UK, in the year prior to MP-AzeFlu initiation (n = 1188). (A) INS: intranasal corticosteroids; OAH: oral anti-histamine; ED: eye drops (∗including chromones, anti-histamines, corticosteroids, ocular lubricants, antibiotics and astringents); non-steroidal NS: nasal spray (†including anti-histamines, anti-cholinergics, chromones, leukotriene receptor antagonists [with diagnosis of rhinitis on the same day]), decongestants and saline).(B) SABA: short-acting β_2_-agonist; ICS: inhaled corticosteroid; LABA: long-acting β_2_-agonist; LTRA: leukotriene receptor antagonist; SAMA: short-acting muscarinic antagonist; NaCrGly: sodium cromoglycate

Multi-morbidity was evident. The top 3 most common co-morbidities recorded (in addition to AR and asthma) were depression/anxiety (n = 479; 40.3%), eczema (ever: n = 370 [31.1%]; active: n = 71 [6.0%]), and chronic rhinosinusitis (CRS; n = 314; 26.4%; Fig. 3). Other less commonly recorded co-morbidities (but still >10%) included hypertension, gastroesophageal reflux disease (GERD; ever), and nasal polyps (NP; Fig. 3). Indeed, 63 patients (5.3%) suffered from both CRS and NP (CRSwNP) (in addition to AR and asthma). The most common AR treatments used prior to MP-AzeFlu prescription were INS and OAHs (Fig. 4A). Over a quarter of patients (n = 319; 26.9%) were prescribed eye drops, and of those patients, most used chromones (n = 111/319; 34.8%). A non-steroidal nasal spray was used by 14.1% of patients (n = 167), with saline nasal spray being the most common in this subset (n = 84/167; 50.3%) (Fig. 4A). Rhinitis poly-pharmacy was common. Over half of patients (n = 612; 51.5%) were prescribed ≥2 rhinitis treatments in the year prior to MP-AzeFlu initiation (Fig. 4A).

#### Baseline asthma clinical characteristics and treatment

Patients in this study had asthma of relatively late onset (although the standard deviation [SD] was large) and of long duration (Table 2). Although the % predicted peak expiratory flow (PEF) and forced expiratory volume in 1 s (FEV_1_) were 77.3% and 85.1%, respectively, at baseline, many patients did show evidence of obstruction; over 1/3 of them (36.6%; n = 56/153) had a % predicted FEV_1_ <80% (S-Table 1) and 29.0% (n = 67/231) had a post-bronchodilator FEV_1_/forced vital capacity (FVC) < 0.7. Severe asthma was noted in 13.1% (n = 156) of patients (defined as those on GINA Step 5 treatment or with uncontrolled asthma on GINA Step 4) and over a quarter of patients had ≥2 asthma consultations (excluding annual review visit) in the year prior to MP-AzeFlu prescription (Table 2). Furthermore, 45.8% (n = 544) of patients had ≥1 acute respiratory event, and 37.7% (n = 448) had ≥1 asthma exacerbation. Only 9.6% (n = 58/604) of patients had controlled asthma (GINA definition) in the baseline year (Table 3).

**Table 2:** Baseline characteristics of patients with allergic rhinitis and asthma before initiation of MP-AzeFlu (n = 1188).

Variable		Variable	
**Age, years, Mean (SD)**	46.8 (19.0)	**Age of asthma onset**^a^**, yrs, Mean (SD) (n = 619)**	24.5 (18.4)
12-17, n (%)	113 (9.5)	**Duration of asthma**^b^**, yrs, Mean (SD) (n = 619)**	22.6 (12.8)
18-64, n (%)	830 (69.9)	**BEC**^c^**, Mean (SD) (n = 921)**	0.3 (0.4)
≥65, n (%)	245 (20.6)	<0.25, n (%)	433 (47.0)
		≥0.25, n (%)	488 (53.0)
**Sex**		**PEF**^d^**, % predicted, Mean (SD) (n = 401)**	77.3 (19.7)
Women, n (%)	694 (58.4)	**FEV**_**1**_^e^**, % predicted, Mean (SD) (n = 153)**	85.1 (22.6)
**Smoking status, ever (n = 1163)**		**FEV**_**1**_**,** ≥ **80% predicted, n (%) (n = 153)**	97.0 (63.4)
Never, n (%)	475 (40.8)	**FEV**_**1**_**/FVC**^e^**, Mean (SD) (n = 231)**	0.8 (0.1)
Current, n (%)	150 (12.9)	**FEV**_**1**_**/FVC**^e^ < **0.7, n (%)**	67.0 (29.0)
Former, n (%)	530 (46.3)		
**BMI, kg/m2 (n = 1038), Mean (SD)**	27.6 (6.3)	**Severe asthma**^f^**, n (%)**	156 (13.1)
Underweight: <18.5, n (%)	50 (4.8)	**Daily dose, μg/day mean (SD)**	
Normal weight: 18.5-<25, n (%)	328 (31.6)	SABA^g^	325.3 (387.8)
Overweight: 25-<30, n (%)	353 (34.0)	ICS^g^	303.9 (386.6)
Obese: ≥30, n (%)	307 (29.6)	**Asthma related consultations,**^h^**n(%)**	
		0	331 (27.9)
		1	521 (43.9)
		≥2	336 (28.3)

SD: standard deviation; BMI: body mass index; BEC: blood eosinophil count; PEF: peak expiratory flow; FEV_1_: forced expiratory flow in 1 s; FVC: forced vital capacity; ICS: inhaled corticosteroid; SABA: short-acting β_2_-agonist.

a Age of asthma onset was determined as when patients had their first diagnostic code for asthma either:•1 year after the date of joining the general practice, and when they did not receive a prescription of asthma medication in that year OR. •Before the date when they joined the general practice and when they did not receive a prescription of asthma medication before they joined the practice.

b Time between the date of MP-AzeFlu initiation and the date of the first diagnostic code for asthma in years.

c No steroid use 2 weeks prior to measurement (10^9^/L);

d Last recorded values closest to index date prior to 5 years (>18 yrs old) or last recorded value closest to index date prior to 2 years (15–18 yrs old);

e Last recorded value closes to IPD prior to 5 years.

f Receiving GINA treatment step 4 plus ≥ 2 exacerbations in baseline year or receiving GINA Step 5 treatment in the baseline year;

g Based on collected prescriptions in the baseline year, salbutamol or fluticasone propionate equivalent.

h An asthma-related consultation but not for annual monitoring/review

**Table 3:** Asthma-related outcomes in the period before and after initiation of the combination therapy azelastine hydrochloride/fluticasone propionate among patients with allergic rhinitis and asthma multi-morbidity (n = 1188).

Variable		Pre-initiation MP-AzeFlu	Post-initiation MP-AzeFlu	Change in outcomes	Effect^a^	P^b^
**Primary outcome**						
Acute respiratory events, number	0, n (%)	644 (54.2)	705 (59.4)	Stable: 632 (53.2%)	5.8%	0.0129
	1, n (%)	267 (22.5)	235 (19.8)	Improved: 312 (26.3%)		
	2, n (%)	130 (10.9)	118 (9.9)	Worsened: 244 (20.5%)		
	3, n (%)	76 (6.4)	48 (4.0)			
	≥4, n (%)	71 (6.0)	82 (6.9)			
**Secondary outcome**						
Asthma exacerbations based on ATS/ERS Force definition, number	0, n (%)	740 (62.3)	783 (65.9)	Stable: 708 (59.6%)	2.4%	0.3545
	1, n (%)	248 (20.9)	201 (16.9)	Improved: 254 (21.4%)		
	2, n (%)	100 (8.4)	107 (9.0)	Worsened: 226 (19.0%)		
	3, n (%)	42 (3.5)	28 (2.4)			
	≥4, n (%)	58 (4.9)	69 (5.8)			
**Exploratory outcomes**						
GINA treatment step	1, n (%)	121 (10.2)	192 (16.2)	Stable: 928 (78.1%)	4.7%	0.0007
	2, n (%)	269 (22.6)	212 (17.8)	Improved: 158 (13.3%)		
	3, n (%)	163 (13.7)	146 (12.3)	Worsened: 102 (8.6%)		
	4, n (%)	635 (53.5)	634 (53.4)			
	5, n (%)	0 (0.0)	<5^c^			
Risk Domain Asthma Control^d^	Controlled, n (%)	620 (52.2)	672 (56.6)	Stable: 864 (72.7%)	4.4%	0.0045
				Improved: 188 (15.8%)		
				(11.4%)		
				Worsened: 136		
Overall Asthma Control^e^	Controlled, n (%)	352 (29.6)	401 (33.8)	Stable: 913 (76.9%)	4.1%	0.0037
				Improved: 162 (13.6%)		
				Worsened: 113 (9.5%)		
GINA Control Status^f^	N (% non-missing)	604 (50.8)	604 (50.8)	Stable: 488 (80.8%)	1.6%	0.3532
	Controlled, n (%)	58 (9.6)	69 (11.4)	Improved: 63 (10.4%)		
	Partly-controlled, n (%)	529 (87.6)	517 (85.6)	Worsened: 53 (8.8%)		
	Uncontrolled, n (%)	17 (2.8)	18 (3.0)			
Average daily dose of SABA based on collected prescriptions, salbutamol equivalent in μg/day	0, n (%)	184 (15.5)	276 (23.2)	Stable: 518 (43.6%)	11.2%	<0.0001
	1-100, n (%)	160 (13.5)	120 (10.1)	Improved: 401 (33.8%)		
	101-200, n (%)	276 (23.2)	227 (19.1)	Worsened: 269 (22.6%)		
	201-300, n (%)	135 (11.4)	171 (14.4)			
	301-400, n (%)	120 (10.1)	121 (10.2)			
	>400, n (%)	313 (26.3)	273 (23.0)			
>2 puffs of SABA per week	Yes, n (%)	1051 (88.5)	958 (80.6)	Stable: 987 (82.5%)	7.7%	<0.0001
				Improved: 147 (12.6%)		
				Worsened: 54 (4.9%)		
Average daily dose of ICS	0, n (%)	141 (11.9)	213 (17.9)	Stable: 761 (64.0%)	4.8%	0.0078
based on collected	>0-≤250, n (%)	563 (47.4)	471 (39.7)	Improved: 242 (20.4%)		
prescriptions, FP	>250-≤500, n (%)	266 (22.4)	286 (24.1)	Worsened: 185 (15.6%)		
equivalent in μg/day	>500, n (%)	218 (18.3)	218 (18.3)			

Abbreviations: MP-AzeFlu, azelastine hydrochloride/fluticasone propionate; ATS/ERS: American Thoracic Society/European Respiratory Society; SABA, short-acting beta agonist; ICS, inhaled corticosteroids; FP, fluticasone propionate; SMD, standardised mean difference.

Controlled = none of the questions have a “yes” response; Partly controlled = 1–2 of the questions have a “yes” response; Uncontrolled = 3–4 of the questions have a “yes” response.

a The effect of the intervention was expressed as the difference in the percentage of patients improving and worsening on a certain asthma outcome

b P-value for the Wilcoxon signed-ranks test (categorical variables), or the McNemar's test (dichotomous variables), where appropriate.

c Data suppressed to comply with privacy requirements (less than a count of 5 in a cell).

d Risk Domain asthma control (RDAC) (yes/no), defined as absence of any of the following events in the baseline year. 1. Acute respiratory event (primary outcome as defined above), *and*. 2. Asthma-related outpatient department (specialist) consultation.

e Overall asthma control (OAC) (yes/no), defined as absence of any of the following events in the baseline year: 1. Acute respiratory event (primary outcome), *and* 2. Asthma-related outpatient department (specialist) consultation, *and* 3. Average daily dose of SABA >200 μg salbutamol/>500 μg terbutaline.

f GINA control status: poor asthma symptom control is defined as 3 out of 4 of the following: 1) “yes” to 3 RCP questions. 2) >2 puffs of SABA per week

In terms of asthma treatment, 53.5% (n = 635) of patients were on GINA step 4 in the baseline year (Table 3); the most common prescriptions were SABA, ICS/long-acting β_2_-agonist (LABA), ICS, and leukotriene receptor antagonist (LTRA) (Fig. 4B). Therapy add-ons to ICS treatment were also prescribed (notably long-acting muscarinic antagonist [LAMA]: 7.7%, n = 92; and LTRA: 22.7%, n = 270). The average daily dose of ICS (FP equivalent in μg/day) was high (303.9 μg [SD 386.6]), with 40.7% of patients (n = 484) prescribed a dose of >250 μg/day. Mean daily SABA dose was also high (325 μg [SD 387.8]), as was acute OCS use, with 35.8% (n = 425) of patients having at least 1 acute OCS prescription in the baseline year (Fig. 4B). Despite this relatively high steroid burden (both inhaled and oral), blood eosinophil count (BEC) was ≥0.25 cells/μl (x 10^9^ cells) for 53% (n = 488/921) of patients (Table 2). Macrolides were prescribed (at least once) to 22.6% (n = 268) of patients during the baseline year (Fig. 4B). A total of 43 patients (3.6%) had ≥3 prescriptions of macrolides in the year prior to MP-AzeFlu prescription.

### Asthma outcomes (year after MP-AzeFlu prescription)

#### Main analysis

Patients with AR had a significant reduction of acute respiratory events during the study period (p = 0.0129), with an effect size of 5.8% more patients improving than worsening. Patients with AR and asthma had better asthma control assessed by RDAC (effect size = 4.4%; p = 0.0045), OAC (effect size = 4.1%; p = 0.0037) and by GINA control status in the year after MP-AzeFlu prescription compared with the year prior to prescription, although the latter association did not reach statistical significance (effect size = 1.6%; p = 0.3532) (Table 3). Although the number of asthma exacerbations did not significantly reduce in the year after MP-AzeFlu initiation, there were 2.4% more patients with fewer exacerbations than there were with more. All of these improvements occurred in an environment of stable or reduced GINA treatment step (for 91.4% of patients), SABA daily dose (11.2% more patients reduced their dose than increased), and ICS daily dose (stable or increased in 84.4% of patients) (Table 3).

#### Supplementary analysis

Although findings were in a similar direction when corrected for ICS, they were non-significant (Online Supplement S-Table 2). Asthma outcomes for those with and without a prescription of INS in the 45 days prior to MP-AzeFlu initiation (Online Supplement S-Table 3A and 3B), or for those who had ever or never been prescribed an INS prior to IPD (Online Supplement S-Tables 4A & 4B) were almost consistent with the main analysis. In patients with an INCS in the 45 days prior to MP-AzeFlu initiation, opposite effects for the number of exacerbations and OAC were observed, but with less precision due to the small sample size. For patients with 0 or 2 exacerbations in the year prior to MP-AzeFlu initiation, almost all asthma outcomes were consistent with the findings of the main analysis, with the exception of number of exacerbations. In this study population, there were 1.4% more patients with more exacerbations than there were with fewer exacerbations, but the number of patients with 0 exacerbations in the year prior to MP-AzeFlu initiation were higher in this study population (68.0% pre-initiation of MP-AzeFlu and 70.4% post-initiation) than in the main analysis (Online Supplement S-Table 5). Finally, the impact of MP-AzeFlu on asthma outcomes was more apparent in those patients with a BEC <0.25 10^9^/L (vs ≥ 0.25 10^9^/L; Online Supplement S-Tables 6A & 6B). However, in the high eosinophilic group MP-AzeFlu was still associated with a significant reduction in SABA use (both in terms of a reduction in average daily dose and requirement for > 2 puffs/week) (Online Supplement S-Table 6B).

## Discussion

This study is the first to show an association between MP-AzeFlu use for AR and improvement in multiple, clinically-relevant, and validated asthma outcomes in real-life clinical practice. MP-AzeFlu use was associated with fewer acute respiratory events and better asthma control in patients with AR and asthma, with a small number of asthma exacerbations prior to MP-AzeFlu initiation. Perhaps more importantly, these benefits occurred despite stable/reduced ICS dose, and in asthma characterized by high treatment (including acute OCS use) and co=morbidity burdens, and was also associated with reduced SABA use. These patients were also frequently treated with multiple AR therapies prior to MP-AzeFlu initiation. Furthermore, asthma benefits were noted in those previously treated with INS (either recently, or ever) and in those with a history of asthma exacerbations in the year prior to MP-AzeFlu initiation. Although, asthma outcomes did not improve in all patients, considering that AR and asthma are among the most prevalent chronic diseases in the world, carrying a high symptomatic and economic burden,^22^^,^^39^, ^40^, ^41^ any indication of therapeutic improvement is welcome.

The positive effect of INS in improving asthma outcomes in asthma and AR co-morbid patients is well-established.^17^^,^^18^^,^^20^^,^^21^ However, it is worth noting that some studies failed to show an effect.^42^, ^43^, ^44^ This may have been because asthma was relatively mild or well-treated, leaving little room for improvement.^42^^,^^43^ Additionally, these studies were of relatively short duration (4–6 weeks), which likely provides insufficient time in which to capture less frequent (or intermittent) evidence of poorly controlled asthma (eg,. exacerbations).^42^^,^^43^ Study size may also have been a factor, leading to a lack of power to detect an effect.^44^ These limitations did not apply to our study. Patients included in the current study had difficult/severe to manage asthma, with plenty of room for improvement. Many patients exhibited irreversible airway obstruction, multiple exacerbations, and poor control, despite evidence of ICS/LABA and acute OCS prescriptions. The patients frequently suffered from other co-morbid conditions in addition to AR, such as CRS, NP, and eczema, and tended to have late onset asthma. High co=morbidity burden and late onset are features of severe asthma,^45^ and indeed 13.1% of patients in our study had a confirmed diagnosis of severe asthma. This is likely an under-estimation; a recent analysis from the OPCRD showed that the majority of patients with severe asthma in primary care are ‘hidden’, and not referred to specialist care for a confirmed diagnosis.^46^ These factors may explain, in part, why MP-AzeFlu did not significantly improve all asthma outcomes investigated (eg, exacerbation rate and GINA control status). However, it is worth noting that although the study was not powered to detect a significant difference in these exploratory outcomes, AR and asthma patients treated with MP-AzeFlu did show a significant improvement in asthma control when assessed using both the RDAC and the OAC. Our study was also of sufficient duration and size to study the impact of MP-AzeFlu on asthma outcomes. A total of 1188 AR and asthma co=morbid patients provided at least 1 full year of data prior to, and after, MP-AzeFlu initiation (which also minimized seasonal bias).

Two previously published socioeconomic studies have provided indirect evidence of MP-AzeFlu's potential to improve asthma control (as a consequence of effectively treating AR).^28^^,^^47^ The first of these, a retrospective claims study for commercially insured patients in the United States, compared MP-AzeFlu with the free combination of intranasal FP plus intranasal azelastine, on healthcare resource utilization and costs for patients with AR and asthma.^28^ It found that asthma-related costs were lower for MP-AzeFlu users (versus those who used the loose combination). Lower asthma pharmacy costs (on MP-AzeFlu), implies less asthma medication usage (also found in our study), which is a surrogate marker of improved asthma control. A second socioeconomic study using Danish National Prescription and Patient Registries data confirmed no increase in asthma medication use for patients on MP-AzeFlu to treat their AR.^47^ Conversely, a significant increase in asthma medication use was noted for patients using concurrent INS + OAH therapy.^47^

Stronger evidence has been forthcoming from a 2-week, prospective, non-interventional study which evaluated the effectiveness of MP-AzeFlu in controlling AR in different AR phenotypes (including patients with AR + asthma, and those with multi-morbidities).^48^^,^^49^ Effectiveness was assessed using a visual analogue scale (VAS). The study showed that 76.5% and 72.8% of multi-morbid and asthma co-morbid patients, respectively, responded to MP-AzeFlu (where response was defined as ≥1 VAS score <50/100 mm), and experienced a rapid, statistically significant, and sustained improvement in AR control in the first days of treatment.^49^ Improvement in AR control was associated with a significant (p ≤ 0.002) 46.2% reduction in asthma VAS score from baseline in patients with AR and asthma. Furthermore, over two-thirds of these co-morbid patients (69.2%) treated with MP-AzeFlu were able to reduce, or considerably reduce, their asthma rescue medication use.^49^ The findings from our study show a similar MP-AzeFlu-associated reduction in SABA use, but also an improvement in many other clinically-relevant and validated asthma outcomes, including asthma control, reduction in GINA treatment step, number of acute respiratory events and ICS dose.

What are the mechanisms underlying MP-AzeFlu's beneficial effect on asthma outcomes? In order for any nasally applied medication to have an effect on the lower airways, it is necessary that the nose and the lungs “talk” to each other (i.e. naso-bronchial cross talk). This phenomenon has been elegantly described by Braunstahl and colleagues.^50^ They found that nasal allergen provocation induced an increase in adhesion molecule expression (eg,. ICAM-1, VCAM-1) and tissue eosinophilia in both the upper and lower airway.^50^ With this channel of communication “open”, an AR medication could exert an anti-asthma effect by: reducing bronchial hyperresponsiveness via re-establishment of primary nasal functions (ie, air filtration and humidification); via an interaction with the bronchial reflex mechanism; and by targeting inflammatory cells and mediators common to both diseases (eg, the eosinophil and its associated cytokines).^2^^,^^3^ MP-AzeFlu has been shown to inhibit eosinophil survival and reduce IL-6 concentrations better than either FP or AZE in a human nasal epithelial cell model, an effect mediated via induction of anti-inflammatory gene expression (ie, GILZ & MKP-1).^51^^,^^52^ This MP-AzeFlu-induced anti-eosinophilic effect was also apparent in the lower airways, evidenced by total abrogation of eosinophils in bronchoalveolar lavage fluid from mice.^53^ Further work is necessary to establish if nasally applied AR medications have a systemic anti-inflammatory effect. It is interesting to note; however, that surgical resection of NP coincides with a reduction in blood eosinophil count in patients with CRS.^54^

The results of our study have implications for the management of asthma and AR co=morbid patients and supports ARIA's recommendation to treat the respiratory tract (both upper and lower) as a whole using a unified treatment approach.^8^ AR patients should be assessed for the presence of asthma by history, and if needed confirmed by reversibility to short-acting β_2_-agonist.^8^ Conversely, patients with uncontrolled asthma, should be assessed for the presence of AR,^3^ since asthma patients with significant rhinitis are nearly 5 times more likely to have poorly controlled asthma (compared to those without rhinitis), with an odds ratio greater than that for poor compliance with asthma therapy.^9^ A combination of the most effective symptomatic treatment for AR (ie, MP-AzeFlu), and appropriate inhaled asthma therapy should be considered in patients with AR and asthma.

Limitations of the current study include a reliance on prescription data as a surrogate for actual medication use. This may have resulted in an over-estimation of MP-AzeFlu use, and an under-estimation of its effect on asthma outcomes. OPCRD does not capture information on rhinitis classification, severity, phenotype, or sensitization patterns which may have provided further insight into the impact of MP-AzeFlu on asthma outcomes, particularly asthma exacerbations. Additional sub-analyses stratified by gender, age, baseline asthma severity and presence of nasal polyps would also have been interesting but insufficient sample size precluded meaningful assessment. OPCRD also does not capture over-the-counter (OTC) medication use. Although all potential MP-AzeFlu use is captured (as MP-AzeFlu is only available on prescription in the United Kingdom), it is possible that patients used other OTC AR medications too. Finally, while we attempted to address confounding factors such as ICS use (the findings were similar to those of the main analysis), recent and ever INS use, and asthma exacerbation history, it is possible (as with all observational studies) that unrecognized confounding factors affected asthma outcomes. To counterbalance these limitations, it should be noted that, by virtue of its size, the OPRCD enabled us to study a large cohort of patients with AR and asthma (>1000 patients), treated with MP-AzeFlu in primary care in the United Kingdom. The pre-post study design allowed patients to act as their own control. Data captured into the OPCRD comes from electronic medical records and has been used frequently for observational research.^13^^,^^14^^,^^55^ These data provide a snapshot of how these patients are managed in real-life in practices all over the United Kingdom, and enabled us to answer important research questions, for which an RCT is unsuitable or unfeasible (due to cost and sample size requirements, for example). Although RCTs are currently considered as the gold-standard study design to determine a cause and effect relationship, they are primarily designed to answer regulatory questions, include a highly selected and homogenous patient population and so seldom represent the wider spectrum of patients seen in clinical practice. The OPCRD data presented in the current study are more heterogeneous in nature (than RCTs), are obtained without RCT supports (eg, free medication, compliance checks, etc) and restrictions (eg, strict inclusion/exclusion criteria) and so are more generalizable to patients seeking medical care in real life.^30^ Finally, by virtue of the number of disease-specific variables collected by OPRCD, we were able to assess the impact of MP-AzeFlu on a comprehensive list of asthma-related outcomes.

In conclusion, our study shows the beneficial effect of MP-AzeFlu (prescribed for the treatment of AR) on asthma outcomes in patients with both AR and asthma in primary care in the United Kingdom. These results are noteworthy since they were obtained in patients seen in real-life clinical practice, and in asthma that was (apparently) well-treated, but remained uncontrolled. Our findings endorse Allergic Rhinitis and its Impact on Asthma (ARIA) and primary care guideline recommendations to treat both the upper and lower airways together,^8^^,^^15^ and serve as a call to action to always check for rhinitis in asthmatic patients. In patients with AR and asthma, getting the upper airway under control may be just as important as controlling other factors which impact asthma control (eg, adherence, inhaler technique). These results should be validated by directly comparing the impact of MP-AzeFlu and INS alone on asthma outcomes in patients with AR and asthma in randomized controlled trials, real-world databases and pragmatic trials, and to quantify the socioeconomic benefit of MP-AzeFlu-associated asthma benefits both directly (eg, reduced healthcare resource utilization) and indirectly (eg, absenteeism/presenteeism).

## Author contact details

Hilda J. I. DE JONG, PhD Observational and Pragmatic Research Institute, Singapore

Jaco VOORHAM, PhD Observational and Pragmatic Research Institute, Singapore

Glenis K. SCADDING, MD Royal National Throat, Nose and Ear Hospital, University College London School of Medicine, London, UK

Claus BACHERT, MD Ghent University Hospital, Ghent, Belgium.

Giorgio Walter CANONICA, MD Personalized Medicine Asthma & Allergy Clinic, Humanitas University & Research Hospital, Milan, Italy, & SANI-Severe Asthma Network Italy.

Peter SMITH, MD Griffith University, Southport, QLD, Australia.

Ulrich WAHN, MD Charité Medical University, Berlin, Germany.

Dermot RYAN, MD Usher Institute, University of Edinburgh, Edinburgh, UK Optimum Patient Care, Cambridge, UK.

Jose A. CASTILLO, MD Hospital Universitari Quirón Dexeus, Barcelona, Spain.

Victoria A. CARTER, BSc Optimum Patient Care, Cambridge, UK

Ruth B. MURRAY, PhD Optimum Patient Care, Cambridge, UK.

David B. PRICE, FRCGP Observational and Pragmatic Research Institute, Singapore; Optimum Patient Care, Cambridge, UK; Academic Primary Care, University of Aberdeen, Aberdeen, UK.

## Study funding

This study was supported by funding from BGP Products Operations GmbH (A MylanCompany). BGP Products Operations GmbH was given the opportunity to review the manuscript for medical and scientific accuracy as well as for intellectual property considerations.

## Author contributions

HJIDJ, JV, VC, RM and DP contributed to the design of the work, analysis and interpretation of data, drafting of the manuscript and manuscript revisions.

GS, CB, GWC, PS, UW, DR and JC contributed to interpretation of data, and revising the article critically for important intellectual content and clinical relevancy.

All authors have approved the final version to be published and to be accountable for all aspects of the work in ensuring that questions related to the accuracy or integrity of any part of the work are appropriately investigated and resolved.

## Declaration of interests

**Hilda J.I. De Jong, Jaco Voorham, and Victoria A. Carter** are employees of the Observational and Pragmatic Research Institute (OPRI).

**Glenis K.Scadding** has received funding for talks and for advice from Mylan, GSK, ALK-Abello, Sanofi and Stallergenes. She chaired the BSACI Rhinitis Guidelines and chairs the EAACI Ethics Committee.

**Claus Bachert** has received fees for lectures, advisory board meetings or search grants from Sanofi-Aventis, Novartis, GSK, Astra-Zeneca, Genzyme, Regeneron, Mylan, Uriach, ALK, Asit Biotech and Stallergenes.

**Giorgio Walter Canonica** has received research grants, as well as lecture or advisory board fees from A. Menarini, Alk-Abello, Allergy Therapeutics, Anallergo, AstraZeneca, MedImmune, Boehringer Ingelheim, Chiesi Farmaceutici, Circassia, Danone, Faes, Genentech, Guidotti-Malesci, GlaxoSmithKline, Hal Allergy, Merck, MSD, Mundipharma, Novartis, Orion, Sanofi-Aventis, Sanofi, Genzyme/Regeneron, Stallergenes, UCB Pharma, Uriach Pharma, Teva, Thermo Fisher, and Valeas.

**Peter Smith** has received an investigator initiated grant and funding for talks and advice from Mylan, has done talks for GSK, AZ, Novartis, Bayer, and Abbott, and has been on advisory boards for Nestle and Seqirus.

**Ulrich Wahn** received honoraria for consultation and lectures from ALK, Stallergenes, Allergopharma, Novartis, Sanofi-Aventis, Roxall, Pfizer, UCB, Biomay, Berlin-Chemie.

**Dermot Ryan** has (in the last 3 years) lectured on behalf of, received sponsorship from, or acted as a paid advisor to Mylan, AZ, Chiesi, Novartis, GSK, Boehringer Ingelheim and Regeneron.

**Jose A. Castillo** reports receipt of fees for lectures, advisory board meetings or research grants from MSD, ALK, AstraZeneca, Boehringer-Ingelheim, Uriach, GSK, Leti and ALK**.** He has chaired the Normativa SEPAR on Asthma, Rhinitis and Nasal Polyp Multimorbidities and chairs The Rhinitis Committee in Asthma Area from SEPAR.

**Ruth B. Murray** reports no conflict of interest.

**David B. Price** has board membership with Amgen, AstraZeneca, Boehringer Ingelheim, Chiesi, Circassia, Mylan, Mundipharma, Novartis, Regeneron Pharmaceuticals, Sanofi Genzyme, Teva Pharmaceuticals, Thermofisher; consultancy agreements with Amgen, AstraZeneca, Boehringer Ingelheim, Chiesi, GlaxoSmithKline, Mylan, Mundipharma, Novartis, Pfizer, Teva Pharmaceuticals, Theravance; grants and unrestricted funding for investigator-initiated studies (conducted through Observational and Pragmatic Research Institute Pte Ltd) from AstraZeneca, Boehringer Ingelheim, Chiesi, Circassia, Mylan, Mundipharma, Novartis, Pfizer, Regeneron Pharmaceuticals, Respiratory Effectiveness Group, Sanofi Genzyme, Teva Pharmaceuticals, Theravance, UK National Health Service; payment for lectures/speaking engagements from AstraZeneca, Boehringer Ingelheim, Chiesi, Cipla, GlaxoSmithKline, Kyorin, Mylan, Mundipharma, Novartis, Regeneron Pharmaceuticals, Sanofi Genzyme, Teva Pharmaceuticals; payment for the development of educational materials from Mundipharma, Novartis; payment for travel/accommodation/meeting expenses from AstraZeneca, Boehringer Ingelheim, Mundipharma, Mylan, Novartis, Thermofisher; funding for patient enrolment or completion of research from Novartis; stock/stock options from AKL Research and Development Ltd which produces phytopharmaceuticals; owns 74% of the social enterprise Optimum Patient Care Ltd (Australia and UK) and 74% of Observational and Pragmatic Research Institute Pte Ltd (Singapore); 5% shareholding in Timestamp which develops adherence monitoring technology; is peer reviewer for grant committees of the Efficacy and Mechanism Evaluation programme, and Health Technology Assessment; and was an expert witness for GlaxoSmithKline.

## Ethics, consent, and permissions

OPCRD operates a general practice “opt-in” and patient “opt-out” system. GP practices choose to contribute de-identified patient data to OPCRD for all patients, with the exception of those who have opted-out from the sharing of their de-identified patient record.

OPCRD has research ethics approval from the UK Health Research Authority (HRA) Research Ethics Committee (REC) to receive and supply patient data for purely observational public health research. Observational research undertaken using OPCRD data must be for public health purposes and approved by the Anonymized Data Ethics and Protocols Transparency (ADEPT) committee. Following ADEPT approval, contractual controls ensure researchers adhere to robust terms and conditions governing data use.

### Consent to publish

The OPCRD is approved by the UK National Health Service for clinical research use (Research Ethics Committee reference: 15/EM/0150). The study protocol was approved by the Anonymized Data Ethics and Protocols Transparency (ADEPT) committee (ADEPT0519). ADEPT is an independent body of experts and regulators commissioned by the Respiratory Effectiveness Group to govern the standard of research conducted on internationally recognised databases.^1^ The study protocol was registered with the European Union electronic Register of Post-Authorization studies (EUPAS30940).

## Submission declaration

This contribution is original. The work has not been published previously and is not currently under evaluation by another journal.

## Availability of data and material

The dataset supporting the conclusions of this article was derived from the Optimum Patient Care Research Database (www.opcrd.co.uk). The OPCRD has ethical approval from the National Health Service (NHS) Research Authority to hold and process anonymized research data (Research Ethics Committee reference: 15/EM/0150). This study was approved by the Anonymized Data Ethics Protocols and Transparency (ADEPT) committee – the independent scientific advisory committee for the OPCRD. The authors do not have permission to give public access to the study dataset; researchers may request access to OPCRD data for their own purposes. Access to OCPRD can be made via the OCPRD website (https://opcrd.co.uk/our-database/data-requests/) or via the enquiries email info@opcrd.co.uk.
