# Supplementary material for: Evaluating the real-life effect of MP-AzeFlu on asthma outcomes in patients with allergic rhinitis and asthma in UK primary care
Source: World Allergy Organ J. 2020 Dec 19;13(12):100490. doi: 10.1016/j.waojou.2020.100490 (PMC7753940; doi:10.1016/j.waojou.2020.100490)
Supplement: Multimedia component 1 [file mmc1.docx]

Online supplementary material:

**Patients**

As MP-AzeFlu is only indicated for AR (both seasonal and perennial) in the UK, it was assumed that patients with a prescription for MP-AzeFlu included in this study had AR. Interestingly, analysis of diagnostic codes ever prior to MP-AzeFlu prescription showed marked overlap of diagnostic codes. This overlap may reflect patients’ journey to and within medical and respiratory care services in the UK resulting in multiple coding/multiple treatment, and/or reflect a co-morbidity burden and represents the ‘real-life’ situation in the UK. Approximately 23% (N=276) of patients had a recording for two different diagnostic codes ever before MP-AzeFlu initiation.

S-Figure 1: overlap of upper respiratory tract diagnostic codes ever prior to MP-AzeFlu initiation.

**Study outcomes: definitions**

| **Co-morbidities** | |
| --- | --- |
| Eczema | - Active (at least on diagnostic Read code for eczema in the baseline year plus a prescription of a topical corticosteroid in the baseline year) - Ever, not active (at least on diagnostic Read code for eczema prior to and including IPD) |
| CRS (ever/never) | - at least one diagnostic Read code for CRS, or by one diagnostic Read code for sinusitis plus at least one prescription of antibiotics on the same day or multiple diagnostic Read codes for sinusitis |
| GERD | - Active (at least on diagnostic Read code for GERD in the baseline year plus a prescription of a GERD drugs in the baseline year) - Ever, not active (at least on diagnostic Read code for GERD prior to and including IPD) |
| **Allergic Rhinitis (AR) treatments** | |
| Non-steroidal NS | - defined by British National Formulary (BNF) section 12.2 (including anti-histamines, decongestants, chromes, ipratropium bromide and saline) |
| Eye drops for allergic conjunctivitis | - defined by BNF section 11 (including anti-histamines, chromones, corticosteroids, tear deficiency, ocular lubricants, antibiotics and astringents) |
| **Asthma Outcomes** | |
| Age of asthma onset | - When patients had their first diagnostic code for asthma ≥1 year after the date when they joined the general practice and when they did not receive a prescription of asthma medication in that year, or - When patients had their first diagnostic code for asthma before the date when they joined the general practice and when they did not receive a prescription of asthma medication before they joined the practice. |
| Duration of asthma | - time between the date of MP-AzeFlu initiation and the date of the first diagnostic code for asthma. |
| BEC | - during steady state of the disease (i.e. no steroid use 2 weeks prior to measurement) closes to IPD within 5 years |
| % predicted PEF | - last recorded value closest to IPD prior to 5 years (>18 years old) or last recorded value closest to IPD prior to 2 years (15-18 years old) - The formula used to calculate PEF in patients aged ≥15 years: - For females: e((0.376*ln(Age))-(0.012*Age)-(58.8/Height)+5.63) - For males: e((0.544*ln(Age))-(0.0151*Age)-(74.7/Height)+5.48) - The formula used to calculate PEF in patients < 15 years:   - 455 x (height/100) - 332 |
| % predicted FEV_1_ | - last recorded value closest to IPD prior to 5 years ≥80% - The formula used to predict FEV_1_:   - For Males: Predicted FEV_1_= (4.30 x height (m)) – (0.029 x age - 2.49)   - For Females: Predicted FEV_1_= (3.95 x height (m)) – (0.025 x age - 2.60) |
| FEV_1_/FVC | - last recorded value closest to IPD prior to 5 years |
| Severe asthma | - patients receiving treatment at Global Initiative for Asthma (GINA) 2018 treatment step 4 and experiencing ≥2 exacerbations, or receiving treatment at GINA step 5. |

| Number of acute respiratory events | - Occurrence of any of the following events separately or together (occurrences within 14 days of each other were considered to belong to the same event):   - Asthma-related primary-care recorded hospital admission^[[1]](#footnote-1)^, *and/or*   - Asthma-related primary-care recorded A&E attendance^[[2]](#footnote-2)^, *and/or*   - Acute course of oral corticosteroids, *and/or*   - Antibiotics course with evidence of respiratory consultation |
| --- | --- |
| Number or asthma exacerbations | - occurrence of any of the following events separately or together, based on the American Thoracic Society/European Respiratory Society (ATS/ERS) Force definition (occurrences within 14 days of each other were considered to belong to the same event): - Asthma-related primary-care recorded hospital admission^[[3]](#footnote-3)^, *and/or* - Asthma-related primary-care recorded A&E attendance^[[4]](#footnote-4)^, *and/or* - Acute course of oral corticosteroids. |
| RDAC | - Absence of all of the following events in the baseline year   - Acute respiratory event (definition described under variable ‘acute respiratory event’), *and*   - Asthma-related outpatient department (specialist) consultation |
| OAC | - the absence of all of the following events in the baseline year:   - Acute respiratory event (definition described under variable ‘acute respiratory event’), *and*   - Asthma-related outpatient department (specialist) consultation, *and*   - Average daily dose of SABA >200 μg salbutamol/ >500 μg terbutaline |
| Asthma treatment | - Based on GINA 2018 guidelines, GINA steps of asthma management in the baseline year |
| Average daily dose of ICS | - based on collected prescriptions in the baseline year, fluticasone propionate (FP) equivalent in μg/day |
| Average daily dose of SABA | - based on collected prescriptions in the baseline year, salbutamol equivalent in μg/day |
| ICS and ICS/LABA adherence | - Medication Possession Ratio (MPR), i.e. the Refill rate (%) = (Total ICS pack days / Number of prescription days) * 100 |
| Asthma related consultations | asthma-related consultation but not for annual monitoring/review. |
| IPD: index prescription date; CRS: chronic rhinosinusitis; GERD: gastroesophageal reflux disease; NS: nasal steroids: PEF: peak expiratory flow; BEC: blood eosinophil count; FEV_1_: forced expiratory volume in one second; FVC: forced vital capacity; A&E: accident & emergency; ATS: American Thoracic Society; ERS: European Respiratory Society; SABA: short-acting β_2_-agonist; RDAC: Domain Asthma Control; OAC: overall asthma control; ICS: inhaled corticosteroid; LABA: long-acting β_2_-agonist | |

**S-Table 1** Baseline lung function

| **Variable** |  |
| --- | --- |
| **PEF % predicted (n=401), mean (SD)**  <50, n (%)  70-47, n (%)  70-79, n (%)  ≥80, n (%) | 77.3 (19.7)  33 (8.2)  95 (23.7)  91 (22.7)  182 (45.4) |
| **FEV_1_ % predicted (n=153), Mean (SD)**  <30, n (%)  30-49, n (%)  50-79, n (%)  ≥80, n (%) | 85.1 (22.6)  <5^a^  6 (3.9)  47 (30.7)  97 (63.4) |
| FEV_1_: forced expiratory volume in one second; PEF: peak expiratory flow rate; SD: standard deviation ^a^Data suppressed to comply with privacy requirements (less than a count of 5 in a cell) | |

**S-Table 2** Asthma-related outcomes in the period before and after initiation of the combination therapy azelastine hydrochloride/fluticasone propionate among patients with allergic rhinitis and asthma multi-morbidity adjusted for inhaled corticosteroids (n=1,188)

| **Outcome** | | **Pre-initiation**  **MP-AzeFlu**  **N = 1,188** | **Post-initiation MP-AzeFlu**  **N = 1,188** | **RR (95% CI)** | **RR (95% CI)** | **P** |
| --- | --- | --- | --- | --- | --- | --- |
| **Primary outcome** | | | | | | |
| Acute respiratory events, number^a,b^ | 0, n (%) | 644 (54.2) | 705 (59.4) | 0.94 (0.86 - 1.03) | 0.95 (0.87 - 1.04) | 0.239 |
|  | 1, n (%) | 267 (22.5) | 235 (19.8) |  |  |  |
|  | 2, n (%) | 130 (10.9) | 118 (9.9) |  |  |  |
|  | 3, n (%) | 76 (6.4) | 48 (4.0) |  |  |  |
|  | 4, n (%) | 23 (1.9) | 27 (2.3) |  |  |  |
|  | 5, n (%) | 26 (2.2) | 18 (1.5) |  |  |  |
|  | ≥6, n (%) | 22 (1.9) | 37 (3.1) |  |  |  |
| **Secondary outcome** | | | | | | |
| Asthma exacerbations based on ATS/ERS Force definition, number^a,b^ | 0, n (%) | 740 (62.3) | 783 (65.9) | 1.01 (0.91 - 1.11) | 1.00 (0.92 - 1.11) | 0.846 |
|  | 1, n (%) | 248 (20.9) | 201 (16.9) |  |  |  |
|  | 2, n (%) | 100 (8.4) | 107 (9.0) |  |  |  |
|  | 3, n (%) | 42 (3.5) | 28 (2.4) |  |  |  |
|  | 4, n (%) | 26 (2.2) | 21 (1.8) |  |  |  |
|  | 5, n (%) | 14 (1.2) | 16 (1.3) |  |  |  |
|  | ≥6, n (%) | 18 (1.5) | 32 (2.7) |  |  |  |
| Gina treatment step^c,d^ | 1, n (%) | 121 (10.2) | 192 (16.2) | Reference | Reference | <0.0001 |
|  | 2, n (%) | 269 (22.6) | 212 (17.8) | 0.25 (0.16 - 0.39) | 0.25 (0.16 - 0.39) |  |
|  | 3, n (%) | 163 (13.7) | 146 (12.3) | 0.29 (0.17 - 0.49) | 0.29 (0.17 - 0.49) |  |
|  | 4, n (%) | 635 (53.5) | 634 (53.4) | 0.42 (0.25 - 0.69) | 0.42 (0.25 - 0.69) |  |
|  | 5, n (%) | 0 (0.0) | <5 | - | - |  |
| Risk Domain Asthma Control^a,e^ | Controlled, n (%) | 620 (52.2) | 672 (56.6) | 1.38 (1.11 - 1.72) | 1.38 (1.11 - 1.72) | 0.004 |
| Overall Asthma Control^a,e^ | Controlled, n (%) | 352 (29.6) | 401 (33.8) | 1.43 (1.13 - 1.82) | 1.43 (1.13 - 1.82) | 0.003 |
| Average daily dose of SABA prescribed, salbutamol equivalent in µg/day^a,b^ | 0, n (%) | 184 (15.5) | 276 (23.2) | 0.75 (0.70 - 0.81) | 0.76 (0.70 - 0.81) | <0.0001 |
|  | 1-100, n (%) | 160 (13.5) | 120 (10.1) |  |  |  |
|  | 101-200, n (%) | 276 (23.2) | 227 (19.1) |  |  |  |
|  | 201-300, n (%) | 135 (11.4) | 171 (14.4) |  |  |  |
|  | 301-400, n (%) | 120 (10.1) | 121 (10.2) |  |  |  |
|  | >400, n (%) | 313 (26.3) | 273 (23.0) |  |  |  |
| GINA control status^a,d^ | N (% non-missing) | 604 (50.8) | 604 (50.8) |  |  |  |
|  | Controlled, n (%) | 58 (9.6) | 69 (11.4) | 1.22 (0.53 - 2.77) | 1.22 (0.53 - 2.77) | 0.8337 |
|  | Partly controlled, n (%) | 529 (87.6) | 517 (85.6) | 0.94 (0.46 - 1.90) | 0.94 (0.46 - 1.90) |  |
|  | Uncontrolled, n (%) | 17 (2.8) | 18 (3.0) | Reference | reference |  |

Abbreviations: MP-AzeFlu, azelastine hydrochloride/fluticasone propionate; RR, rate ratio; OR, Odds ratio

^a^Adjusted for doses of the change in inhaled corticosteroids

^b^Count outcomes: a conditional negative binomial regression was used, and the result were reported as rate ratio (RR) and corresponding 95% confidence interval (CI).

^c^Not adjusted for doses of the change in inhaled corticosteroids

^d^Multinomial outcomes: for outcomes with more than two categories (e.g. Gina step), conditional multinomial logistic regression was used. For this analysis the odds ratio (OR) and 95% CI were presented.

^e^Binary outcomes: a conditional logistic regression was used, and findings were reported as odds ratio (OR) and 95% CI.

**S-Table** **3A** Asthma-related outcomes among patients with allergic rhinitis and asthma who were prescribed intranasal corticosteroids in the 45 days before initiation of MP-AzeFlu (n=283)

| Variable | | Pre-initiation MP-AzeFlu  N = 283 | Post-initiation MP-AzeFlu  N = 283 | Changes in outcomes | Effect^a^ | P^b^ |
| --- | --- | --- | --- | --- | --- | --- |
| **Primary outcome** | | | | | | |
| Acute respiratory events, number | 0, n (%) | 131 (46.3) | 142 (50.2) | Stable: 130 (45.9) | 1.1% | 0.8398 |
|  | 1, n (%) | 69 (24.4) | 67 (23.7) | Improved: 78 (27.6) |  |  |
|  | 2, n (%) | 34 (12.0) | 31 (10.9) | Worsened: 75 (26.5) |  |  |
|  | 3, n (%) | 23 (8.1) | 15 (5.3) |  |  |  |
|  | 4, n (%) | 9 (3.2) | 7 (2.5) |  |  |  |
|  | 5, n (%) | 9 (3.2) | 8 (2.8) |  |  |  |
|  | ≥6, n (%) | 8 (2.8) | 13 (4.6) |  |  |  |
| **Secondary outcome** | | | | | | |
| Asthma exacerbations based on ATS/ERS Force definition, number | 0, n (%) | 158 (55.8) | 160 (56.5) | Stable: 146 (51.6) | -5.4% | 0.1816 |
|  | 1, n (%) | 66 (23.3) | 58 (20.5) | Improved: 61 (21.5) |  |  |
|  | 2, n (%) | 23 (8.1) | 32 (11.3) | Worsened: 76 (26.9) |  |  |
|  | 3, n (%) | 14 (4.9) | 7 (2.5) |  |  |  |
|  | 4, n (%) | 11 (3.9) | 9 (3.2) |  |  |  |
|  | 5, n (%) | 6 (2.1) | 5 (1.8) |  |  |  |
|  | ≥6, n (%) | 5 (1.9) | 12 (4.2) |  |  |  |
| **Exploratory outcomes** | | | | | | |
| GINA treatment step | 1, n (%) | 11 (3.9) | 28 (9.9) | Stable: 230 (81.3) | 3.9% | 0.1276 |
|  | 2, n (%) | 63 (22.3) | 47 (16.6) | Improved: 32 (11.3) |  |  |
|  | 3, n (%) | 37 (13.1) | 38 (13.4) | Worsened: 21 (7.4) |  |  |
|  | 4, n (%) | 172 (60.8) | 169 (59.7) |  |  |  |
|  | 5, n (%) | 0 (0.0) | <5^c^ |  |  |  |
| Risk Domain Asthma Control^d^ | Controlled, n (%) | 128 (45.2) | 131 (46.3) | Stable: 202 (71.4)  Improved: 42 (14.8)  Worsened: 39 (13.8) | 1.0% | 0.8243 |
| Overall Asthma Control^e^ | Controlled, n (%) | 73 (25.8) | 69 (24.4) | Stable: 219 (77.4)  Improved: 30 (10.6)  Worsened: 34 (12.0) | -1.4% | 0.8243 |
| Average daily dose of SABA based on collected prescriptions, salbutamol equivalent in µg/day | 0, n (%) | 35 (12.4) | 60 (21.2) | Stable: 120 (42.4) | 7.4% | 0.1114 |
|  | 1-100, n (%) | 34 (12.0) | 19 (6.7) | Improved: 92 (32.5) |  |  |
|  | 101-200, n (%) | 61 (21.6) | 42 (14.8) | Worsened: 71 (25.1) |  |  |
|  | 201-300, n (%) | 33 (11.7) | 45 (15.9) |  |  |  |
|  | 301-400, n (%) | 23 (8.1) | 28 (9.9) |  |  |  |
|  | >400, n (%) | 97 (34.3) | 89 (31.4) |  |  |  |
| >2 puffs of SABA per week | Yes, n (%) | 248 (87.6) | 223 (78.8) | Stable: 230 (81.3)  Improved: 39 (13.8)  Worsened:14 (4.9) | 8.9% | 0.0008 |
| Average daily dose of ICS | 0, n (%) | 19 (6.7) | 31 (11.0) | Stable: 181 (64.0%) | 5.6% | 0.1262 |
| based on collected | >0-≤250, n (%) | 112 (39.6) | 97 (34.3) | Improved: 59 (20.8%) |  |  |
| prescriptions, FP | >250-≤500, n (%) | 82 (29.0) | 91 (32.2) | Worsened: 43 (15.2%) |  |  |
| equivalent in µg/day | >500, n (%) | 70 (24.7) | 64 (22.6) |  |  |  |
| GINA control status^f^ | N (% non-missing) | 160 (56.5) | 160 (56.5) | Stable: 125 (78.1) | 1.9% | 0.6121 |
|  | Controlled, n (%) | 17 (10.6) | 21 (13.1) | Improved: 19 (11.9) |  |  |
|  | Partly controlled, n (%) | 139 (86.9) | 134 (83.8) | Worsened: 16 (10.0) |  |  |
|  | Uncontrolled, n (%) | <5 | 5 (3.1) |  |  |  |

Abbreviations: MP-AzeFlu, azelastine hydrochloride/fluticasone propionate; ATS/ERS, American Thoracic Society/European Respiratory Society; SABA, short-acting beta agonist; ICS, inhaled corticosteroids; FP, fluticasone propionate

^a^Effect is expressed as % of patients that remained stable or improved from baseline to outcome 1-year periods.

^b^P-value for the Wilcoxon signed-ranks test (categorical variables), or the McNemar’s test (dichotomous variables), where appropriate

^c^Data suppressed to comply with privacy requirements (less than a count of 5 in a cell

^d^Risk Domain asthma control (RDAC) (yes/no), defined as absence of any of the following events in the baseline year

1. Acute respiratory event (primary outcome as defined above), *and*
2. Asthma-related outpatient department visit

^e^Overall asthma control (OAC) (yes/no), defined as absence of any of the following events in the baseline year:

1. Acute respiratory event (primary outcome), *and*
2. Asthma-related outpatient department visit, *and*
3. Average daily dose of SABA >200 μg salbutamol/ >500 μg terbutaline

^f^GINA control status: poor asthma symptom control is defined as 3 out of 4 of the following:

1. “yes” to 3 RCP questions and/or
2. >2 puffs of SABA per week

Controlled = none of the questions have a “yes” response; Partly controlled = 1-2 of the questions have a “yes” response; Uncontrolled = 3-4 of the questions have a “yes” response

**S-Table 3B** Asthma-related outcomes among patients with allergic rhinitis and asthma multi-morbidity who were not prescribed intranasal corticosteroids in the 45 days before initiation of the combination therapy azelastine hydrochloride/fluticasone propionate (n=905)

| Variable | | Pre-initiation MP-AzeFlu  N = 905 | Post-initiation MP-AzeFlu  N = 905 | Changes in outcomes | Effect^a^ | P^b^ |
| --- | --- | --- | --- | --- | --- | --- |
| **Primary outcome** | | | | | | |
| Acute respiratory events, number | 0, n (%) | 513 (56.7) | 563 (62.2) | Stable: 502 (55.5) | 7.1% | 0.0046 |
|  | 1, n (%) | 198 (21.9) | 168 (18.6) | Improved: 234 (25.8) |  |  |
|  | 2, n (%) | 96 (10.6) | 87 (9.6) | Worsened: 169 (18.7) |  |  |
|  | 3, n (%) | 53 (5.9) | 33 (3.6) |  |  |  |
|  | 4, n (%) | 14 (1.5) | 20 (2.2) |  |  |  |
|  | 5, n (%) | 17 (1.9) | 10 (1.1) |  |  |  |
|  | ≥6, n (%) | 14 (1.5) | 24 (2.7) |  |  |  |
| **Secondary outcome** | | | | | | |
| Asthma exacerbations based on ATS/ERS Force definition, number | 0, n (%) | 582 (64.3) | 623 (68.8) | Stable: 562 (62.1) | 4.7% | 0.0470 |
|  | 1, n (%) | 182 (20.1) | 143 (15.9) | Improved: 193 (21.3) |  |  |
|  | 2, n (%) | 77 (8.5) | 75 (8.3) | Worsened: 150 (16.6) |  |  |
|  | 3, n (%) | 28 (3.1) | 21 (2.3) |  |  |  |
|  | 4, n (%) | 15 (1.7) | 12 (1.3) |  |  |  |
|  | 5, n (%) | 8 (0.9) | 11 (1.2) |  |  |  |
|  | ≥6, n (%) | 13 (1.4) | 20 (2.2) |  |  |  |
| **Exploratory outcomes** | | | | | | |
| GINA treatment step | 1, n (%) | 110 (12.2) | 164 (18.1) | Stable: 694 (76.7) | 4.7% | 0.0039 |
|  | 2, n (%) | 206 (22.8) | 165 (18.2) | Improved: 127 (14.0) |  |  |
|  | 3, n (%) | 126 (13.9) | 110 (12.2) | Worsened: 84 (9.3) |  |  |
|  | 4, n (%) | 463 (51.2) | 463 (51.2) |  |  |  |
|  | 5, n (%) | 0 (0.0) | <5^c^ |  |  |  |
| Risk Domain Asthma Control^d^ | Controlled, n (%) | 492 (54.4) | 541 (59.8) | Stable: 662 (73.2)  Improved: 146 (16.1)  Worsened: 97 (10.7) | 5.4% | 0.0020 |
| Overall Asthma Control^e^ | Controlled, n (%) | 279 (30.8) | 332 (36.7) | Stable: 694 (76.7)  Improved: 132 (14.6)  Worsened: 79 (8.7) | 5.9% | 0.0003 |
| Average daily dose of SABA based on collected prescriptions, salbutamol equivalent in µg/day | 0, n (%) | 102 (11.3) | 170 (18.8) | Stable: 398 (44.0) | 12.2% | <0.0001 |
|  | 1-100, n (%) | 119 (13.1) | 100 (11.0) | Improved: 309 (34.1) |  |  |
|  | 101-200, n (%) | 225 (24.9) | 186 (20.6) | Worsened: 198 (21.9) |  |  |
|  | 201-300, n (%) | 110 (12.2) | 137 (15.1) |  |  |  |
|  | 301-400, n (%) | 103 (11.4) | 93 (10.3) |  |  |  |
|  | >400, n (%) | 246 (27.2) | 219 (24.2) |  |  |  |
| >2 puffs of SABA per week | yes, n (%) | 803 (88.7) | 735 (81.2) | Stable: 757 (83.7)  Improved: 108 (11.9)  Worsened: 40 (4.4) | 7.5% | <0.0001 |
| Average daily dose of ICS | 0, n (%) | 122 (13.5) | 182 (20.1) | Stable: 580 (64.1%) | 4.5% | 0.0282 |
| based on collected | >0-≤250, n (%) | 451 (49.8) | 374 (41.3) | Improved: 183 (20.2%) |  |  |
| prescriptions, FP | >250-≤500, n (%) | 184 (20.3) | 195 (21.5) | Worsened: 142 (15.7%) |  |  |
| equivalent in µg/day | >500, n (%) | 148 (16.4) | 154 (17.0) |  |  |  |
| GINA control status^f^ | N (% non-missing) | 444 (49.1) | 444 (49.1) | Stable: 363 (81.8) | 1.6% | 0.4367 |
|  | Controlled, n (%) | 41 (9.2) | 48 (10.8) | Improved: 44 (9.9) |  |  |
|  | Partly controlled, n (%) | 390 (87.8) | 383 (86.3) | Worsened: 37 (8.3) |  |  |
|  | Uncontrolled, n (%) | 13 (2.9) | 13 (2.9) |  |  |  |

Abbreviations: MP-AzeFlu, azelastine hydrochloride/fluticasone propionate; ATS/ERS, American Thoracic Society/European Respiratory Society; SABA, short-acting beta agonist; ICS, inhaled corticosteroids; FP, fluticasone propionate

^a^Effect is expressed as % of patients that remained stable or improved from baseline to outcome 1-year periods.

^b^P-value for the Wilcoxon signed-ranks test (categorical variables), or the McNemar’s test (dichotomous variables), where appropriate

^c^Data suppressed to comply with privacy requirements (less than a count of 5 in a cell)

^d^Risk Domain asthma control (RDAC) (yes/no), defined as absence of any of the following events in the baseline year

1. Acute respiratory event (primary outcome as defined above), *and*
2. Asthma-related outpatient department visit

^e^Overall asthma control (OAC) (yes/no), defined as absence of any of the following events in the baseline year:

1. Acute respiratory event (primary outcome), *and*
2. Asthma-related outpatient department visit, *and*
3. Average daily dose of SABA >200 μg salbutamol/ >500 μg terbutaline

^f^GINA control status: poor asthma symptom control is defined as 3 out of 4 of the following:

1. “yes” to 3 RCP questions and/or
2. >2 puffs of SABA per week

Controlled = none of the questions have a “yes” response; Partly controlled = 1-2 of the questions have a “yes” response; Uncontrolled = 3-4 of the questions have a “yes” response

**S-Table 4A** Asthma-related outcomes among patients with allergic rhinitis and asthma who were ever prescribed intranasal corticosteroids before initiation of MP-AzeFlu (n=1,013)

| Variable | | Pre-initiation MP-AzeFlu  N = 1,013 | | Post-initiation MP-AzeFlu  N = 1,013 | Change in outcomes | Effect^a^ | P^b^ |
| --- | --- | --- | --- | --- | --- | --- | --- |
| **Primary outcome** | | | | | | | |
| Acute respiratory events, number | 0, n (%) | 535 (52.8) | | 590 (58.2) | Stable: 524 (51.7) | 5.5% | 0.0420 |
|  | 1, n (%) | 238 (23.5) | | 208 (20.5) | Improved: 272 (26.9) |  |  |
|  | 2, n (%) | 116 (11.5) | | 101 (10.0) | Worsened: 217 (21.4) |  |  |
|  | 3, n (%) | 65 (6.4) | | 43 (4.2) |  |  |  |
|  | 4, n (%) | 20 (2.0) | | 24 (2.4) |  |  |  |
|  | 5, n (%) | 22 (2.2) | | 16 (1.6) |  |  |  |
|  | ≥6, n (%) | 17 (1.6) | | 31 (3.1) |  |  |  |
| **Secondary outcome** | | | | | | | |
| Asthma exacerbations based on ATS/ERS Force definition, number | 0, n (%) | 620 (61.2) | | 659 (65.0) | Stable: 589 (58.1) | 2.3% | 0.4512 |
|  | 1, n (%) | 220 (21.7) | | 177 (17.5) | Improved: 224 (22.1) |  |  |
|  | 2, n (%) | 89 (8.8) | | 95 (9.4) | Worsened: 200 (19.8) |  |  |
|  | 3, n (%) | 36 (3.5) | | 23 (2.3) |  |  |  |
|  | 4, n (%) | 22 (2.2) | | 19 (1.9) |  |  |  |
|  | 5, n (%) | 13 (1.3) | | 13 (1.3) |  |  |  |
|  | ≥6, n (%) | 13 (1.3) | | 27 (2.6) |  |  |  |
| **Exploratory outcomes** | | | | | | | |
| GINA treatment step | 1, n (%) | | 99 (9.8) | 161 (15.9) | Stable: 793 (78.3) | 4.9% | 0.0008 |
|  | 2, n (%) | | 223 (22.0) | 179 (17.7) | Improved: 135 (13.3) |  |  |
|  | 3, n (%) | | 142 (14.0) | 125 (12.3) | Worsened: 85 (8.4) |  |  |
|  | 4, n (%) | | 549 (54.2) | 545 (53.8) |  |  |  |
|  | 5, n (%) | | 0 (0.0) | <5^c^ |  |  |  |
| Risk Domain Asthma Control^d^ | Controlled, n (%) | | 517 (51.0) | 563 (55.6) | Stable: 725 (71.6)  Improved: 167 (16.5)  Worsened: 121 (11.9) | 4.6% | 0.0079 |
| Overall Asthma Control^e^ | Controlled, n (%) | | 304 (30.0) | 343 (33.9) | Stable: 776 (76.6)  Improved: 138 (13.6)  Worsened: 99 (9.8) | 3.8% | 0.0134 |
| Average daily dose of SABA based on collected prescriptions, salbutamol equivalent in µg/day | 0, n (%) | | 117 (11.5) | 200 (19.7) | Stable: 438 (43.2) | 11.4% | <0.0001 |
|  | 1-100, n (%) | | 132 (13.0) | 103 (10.2) | Improved: 345 (34.1) |  |  |
|  | 101-200, n (%) | | 250 (24.7) | 191 (18.9) | Worsened: 230 (22.7) |  |  |
|  | 201-300, n (%) | | 124 (12.2) | 157 (15.5) |  |  |  |
|  | 301-400, n (%) | | 99 (9.8) | 104 (10.3) |  |  |  |
|  | >400, n (%) | | 291 (28.7) | 258 (25.5) |  |  |  |
| >2 puffs of SABA per week | yes, n (%) | | 896 (88.5) | 813 (80.3) | Stable: 840 (82.9)  Improved: 128 (12.7)  Worsened: 45 (4.4) | 8.3% | <0.0001 |
| Average daily dose of ICS | 0, n (%) | | 117 (11.5) | 178 (17.6) | Stable: 652 (64.4%) | 4.8% | 0.0125 |
| based on collected | >0-≤250, n (%) | | 470 (46.4) | 396 (39.1) | Improved: 205 (20.2%) |  |  |
| prescriptions, FP | >250-≤500, n (%) | | 241 (23.8) | 252 (24.9) | Worsened: 156 (15.4%) |  |  |
| equivalent in µg/day | >500, n (%) | | 185 (18.3) | 187 (18.5) |  |  |  |
| GINA control status^f^ | N (% non-missing) | | 524 (51.7) | 524 (51.7) | Stable: 422 (80.5) | 1.9% | 0.3221 |
|  | Controlled, n (%) | | 48 (9.2) | 59 (11.3) | Improved: 56 (10.7) |  |  |
|  | Partly controlled, n (%) | | 460 (87.8) | 448 (85.5) | Worsened: 46 (8.8) |  |  |
|  | Uncontrolled, n (%) | | 16 (3.1) | 17 (3.2) |  |  |  |

Abbreviations: MP-AzeFlu, azelastine hydrochloride/fluticasone propionate; ATS/ERS, American Thoracic Society/European Respiratory Society; SABA, short-acting beta agonist; ICS, inhaled corticosteroids; FP, fluticasone propionate

^a^Effect is expressed as % of patients that remained stable or improved from baseline to outcome 1-year periods.

^b^P-value for the Wilcoxon signed-ranks test (categorical variables), or the McNemar’s test (dichotomous variables), where appropriate

^c^Data suppressed to comply with privacy requirements (less than a count of 5 in a cell)

^d^Risk Domain asthma control (RDAC) (yes/no), defined as absence of any of the following events in the baseline year

1. Acute respiratory event (primary outcome as defined above), *and*
2. Asthma-related outpatient department visit

^e^Overall asthma control (OAC) (yes/no), defined as absence of any of the following events in the baseline year:

1. Acute respiratory event (primary outcome), *and*
2. Asthma-related outpatient department visit, *and*
3. Average daily dose of SABA >200 μg salbutamol/ >500 μg terbutaline

^f^GINA control status: poor asthma symptom control is defined as 3 out of 4 of the following:

1. “yes” to 3 RCP questions and/or
2. >2 puffs of SABA per week

Controlled = none of the questions have a “yes” response; Partly controlled = 1-2 of the questions have a “yes” response; Uncontrolled = 3-4 of the questions have a “yes” response

**S-Table 4B** Asthma-related outcomes among patients with allergic rhinitis and asthma multi-morbidity who were never prescribed intranasal corticosteroids before initiation with MP-AzeFlu (n=175)

| Variable | | Pre-initiation MP-AzeFlu  N = 175 | Post-initiation MP-AzeFlu  N = 175 | Change in outcomes | Effect^a^ | P^b^ |
| --- | --- | --- | --- | --- | --- | --- |
| **Primary outcome** | | | | | | |
| Acute respiratory events, number | 0, n (%) | 109 (62.3) | 115 (65.7) | Stable: 108 (61.7) | 7.5% | 0.1096 |
|  | 1, n (%) | 29 (16.6) | 27 (15.4) | Improved: 40 (22.9) |  |  |
|  | 2, n (%) | 14 (8.0) | 17 (9.7) | Worsened: 27 (15.4) |  |  |
|  | 3, n (%) | 11 (6.3) | 5 (2.9) |  |  |  |
|  | ≥4, n (%) | 12 (6.8) | 11 (6.3) |  |  |  |
| **Secondary outcome** | | | | | | |
| Asthma exacerbations based on ATS/ERS Force definition, number | 0, n (%) | 120 (68.6) | 124 (70.8) | Stable: 119 (68.0) | 2.2% | 0.5731 |
|  | 1, n (%) | 28 (16.0) | 24 (13.7) | Improved: 30 (17.1) |  |  |
|  | 2, n (%) | 11 (6.3) | 12 (6.9) | Worsened: 26 (14.9) |  |  |
|  | 3, n (%) | 6 (3.4) | 5 (2.9) |  |  |  |
|  | ≥4, n (%) | 10 (5.7) | 10 (5.7) |  |  |  |
| **Exploratory outcomes** | | | | | | |
| GINA treatment step | 1, n (%) | 22 (12.6) | 31 (17.7) | Stable: 132 (75.5) | 1.7% | 0.7001 |
|  | 2, n (%) | 46 (26.3) | 33 (18.9) | Improved: 23 (13.1) |  |  |
|  | 3, n (%) | 21 (12.0) | 22 (12.6) | Worsened: 20 (11.4) |  |  |
|  | 4, n (%) | 86 (49.1) | 88 (50.3) |  |  |  |
|  | 5, n (%) | 0 (0.0) | <5^c^ |  |  |  |
| Risk Domain Asthma Control^d^ | Controlled, n (%) | 103 (58.9) | 109 (62.3) | Stable: 139 (79.4)  Improved: 21 (12.0)  Worsened: 15 (8.6) | 3.4% | 0.4050 |
| Overall Asthma Control^e^ | Controlled, n (%) | 48 (27.4) | 58 (33.1) | Stable: 137 (78.3)  Improved: 24 (13.7)  Worsened: 14 (8.0) | 5.7% | 0.1433 |
| Average daily dose of SABA based on collected prescriptions, salbutamol equivalent in µg/day | 0, n (%) | 20 (11.4) | 30 (17.1) | Stable: 80 (45.7) | 9.7% | 0.0584 |
|  | 1-100, n (%) | 21 (12.0) | 16 (9.1) | Improved: 56 (32.0) |  |  |
|  | 101-200, n (%) | 36 (20.6) | 37 (21.1) | Worsened: 39 (22.3) |  |  |
|  | 201-300, n (%) | 19 (10.9) | 25 (14.3) |  |  |  |
|  | 301-400, n (%) | 27 (15.4) | 17 (9.7) |  |  |  |
|  | >400, n (%) | 52 (29.7) | 50 (28.6) |  |  |  |
| >2 puffs of SABA per week | Yes, n (%) | 155 (88.6) | 145 (82.9) | Stable: 147 (84.0)  Improved: 19 (10.9)  Worsened: 9 (5.1) | 5.8% | 0.0872 |
| Average daily dose of ICS | 0, n (%) | 24 (13.7) | 35 (20.0) | Stable: 109 (62.3%) | 4.5% | 0.3539 |
| based on collected | >0-≤250, n (%) | 93 (53.1) | 75 (42.9) | Improved: 37 (21.1%) |  |  |
| prescriptions, FP | >250-≤500, n (%) | 25 (14.3) | 34 (19.4) | Worsened: 29 (16.6%) |  |  |
| equivalent in µg/day | >500, n (%) | 33 (18.9) | 31 (17.7) |  |  |  |
| GINA control status^f^ | N (% non-missing) | 80 (45.7) | 80 (45.7) | Stable: 66 (82.6) | 0.0% | 1.0000 |
|  | Controlled, n (%) | 10 (12.5) | 10 (12.5) | Improved: 7 (8.7) |  |  |
|  | Partly controlled, n (%) | 69 (86.2) | 69 (86.2) | Worsened: 7 (8.7) |  |  |
|  | Uncontrolled, n (%) | <5 | <5 |  |  |  |

Abbreviations: MP-AzeFlu, azelastine hydrochloride/fluticasone propionate; ATS/ERS, American Thoracic Society/European Respiratory Society; SABA, short-acting beta agonist; ICS, inhaled corticosteroids; FP, fluticasone propionate

^a^Effect is expressed as % of patients that remained stable or improved from baseline to outcome 1-year periods.

^b^P-value for the Wilcoxon signed-ranks test (categorical variables), or the McNemar’s test (dichotomous variables), where appropriate

^c^Data suppressed to comply with privacy requirements (less than a count of 5 in a cell)

^d^Risk Domain asthma control (RDAC) (yes/no), defined as absence of any of the following events in the baseline year

1. Acute respiratory event (primary outcome as defined above), *and*
2. Asthma-related outpatient department visit

^e^Overall asthma control (OAC) (yes/no), defined as absence of any of the following events in the baseline year:

1. Acute respiratory event (primary outcome), *and*
2. Asthma-related outpatient department visit, *and*
3. Average daily dose of SABA >200 μg salbutamol/ >500 μg terbutaline

^f^GINA control status: poor asthma symptom control is defined as 3 out of 4 of the following:

1. “yes” to 3 RCP questions and/or
2. >2 puffs of SABA per week

Controlled = none of the questions have a “yes” response; Partly controlled = 1-2 of the questions have a “yes” response; Uncontrolled = 3-4 of the questions have a “yes” response

**S-Table 5** Asthma-related outcomes among patients with allergic rhinitis and asthma with none to two exacerbations in the year before initiation of MP-AzeFlu (n=1,088)

| Variable | | Pre-initiation MP-AzeFlu  N=1,088 | Post-initiation MP-AzeFlu  N=1,088 | Change in outcomes | Effect^a^ | P^b^ |
| --- | --- | --- | --- | --- | --- | --- |
| **Primary outcome** | | | | | | |
| Acute respiratory events, number | 0, n (%) | 644 (59.2) | 691 (63.5) | Stable: 619 (56.9) | 2.1% | 0.6013 |
|  | 1, n (%) | 267 (24.5) | 219 (20.1) | Improved: 246 (22.6) |  |  |
|  | 2, n (%) | 130 (11.9) | 101 (9.3) | Worsened: 223 (20.5) |  |  |
|  | 3, n (%) | 39 (3.6) | 31 (2.9) |  |  |  |
|  | 4, n (%) | 5 (0.5) | 21 (1.9) |  |  |  |
|  | 5, n (%) | <5^c^ | 12 (1.1) |  |  |  |
|  | ≥6, n (%) | 0 (0.0) | 13 (1.2) |  |  |  |
| **Secondary outcome** | | | | | | |
| Asthma exacerbations based on ATS/ERS Force definition, number | 0, n (%) | 740 (68.0) | 766 (70.4) | Stable: 699 (64.2) | -1.4% | 0.1970 |
|  | 1, n (%) | 248 (22.8) | 187 (17.2) | Improved: 187 (17.2) |  |  |
|  | 2, n (%) | 100 (9.2) | 84 (7.7) | Worsened: 202 (18.6) |  |  |
|  | 3, n (%) | 0 (0.0) | 17 (1.6) |  |  |  |
|  | 4, n (%) | 0 (0.0) | 16 (1.5) |  |  |  |
|  | 5, n (%) | 0 (0.0) | 9 (0.8) |  |  |  |
|  | ≥6, n (%) | 0 (.00) | 9 (0.8) |  |  |  |
| **Exploratory outcomes** | | | | | | |
| GINA treatment step | 1, n (%) | 121 (11.1) | 186 (17.1) | Stable: 852 (78.3) | 5.1% | 0.0003 |
|  | 2, n (%) | 259 (23.8) | 208 (19.1) | Improved: 146 (13.4) |  |  |
|  | 3, n (%) | 151 (13.9) | 139 (12.8) | Worsened: 90 (8.3) |  |  |
|  | 4, n (%) | 557 (51.2) | 555 (51.0) |  |  |  |
| Risk Domain Asthma Control^d^ | Controlled, n (%) | 620 (57.0) | 663 (60.9) | Stable: 773 (71.0)  Improved: 179 (16.5)  Worsened: 136 (12.5) | 4.0% | 0.0178 |
| Overall Asthma Control^e^ | Controlled, n (%) | 352 (32.4) | 396 (36.4) | Stable: 818 (75.2)  Improved: 157 (14.4)  Worsened: 113 (10.4) | 4.0% | 0.0088 |
| Average daily dose of SABA prescribed, salbutamol equivalent in µg/day | 0, n (%) | 133 (12.2) | 223 (20.5) | Stable: 460 (42.3) | 11.7% | <0.0001 |
|  | 1-100, n (%) | 144 (13.2) | 114 (10.5) | Improved: 378 (34.7) |  |  |
|  | 101-200, n (%) | 271 (24.9) | 214 (19.7) | Worsened: 250 (23.0) |  |  |
|  | 201-300, n (%) | 135 (12.4) | 170 (15.6) |  |  |  |
|  | 301-400, n (%) | 116 (10.7) | 107 (9.8) |  |  |  |
|  | >400, n (%) | 289 (26.6) | 260 (23.9) |  |  |  |
| >2 puffs of SABA per week | yes, n (%) | 955 (87.8) | 865 (79.5) | Stable: 896 (82.4)  Improved: 141 (12.9)  Worsened: 51 (4.7) | 8.2% | <0.0001 |
| Average daily dose of ICS | 0, n (%) | 139 (12.8) | 204 (18.8) | Stable: 702 (64.5%) | 5.5% | 0.0031 |
| based on collected | >0-≤250, n (%) | 531 (48.8) | 448 (41.2) | Improved: 223 (20.5%) |  |  |
| prescriptions, FP | >250-≤500, n (%) | 233 (21.4) | 258 (23.7) | Worsened: 163 (15.0%) |  |  |
| equivalent in µg/day | >500, n (%) | 185 (17.0) | 178 (16.4) |  |  |  |
| GINA control status^f^ | N (% non-missing) | 556 (51.1) | 556 (51.1) | Stable: 449 (80.8) | 2.4% | 0.2088 |
|  | Controlled, n (%) | 55 (9.9) | 68 (12.2) | Improved: 60 (10.8) |  |  |
|  | Partly controlled, n (%) | 487 (87.6) | 474 (85.3) | Worsened: 47 (8.4) |  |  |
|  | Uncontrolled, n (%) | 14 (2.5) | 14 (2.5) |  |  |  |

Abbreviations: MP-AzeFlu, azelastine hydrochloride/fluticasone propionate; ATS/ERS, American Thoracic Society/European Respiratory Society; SABA, short-acting beta agonist; ICS, inhaled corticosteroids; FP, fluticasone propionate

^a^Effect is expressed as % of patients that remained stable or improved from baseline to outcome 1-year periods.

^b^P-value for the Wilcoxon signed-ranks test (categorical variables), or the McNemar’s test (dichotomous variables), where appropriate

^c^Data suppressed to comply with privacy requirements (less than a count of 5 in a cell)

^d^Risk Domain asthma control (RDAC) (yes/no), defined as absence of any of the following events in the baseline year

1. Acute respiratory event (primary outcome as defined above), *and*
2. Asthma-related outpatient department visit

^e^Overall asthma control (OAC) (yes/no), defined as absence of any of the following events in the baseline year:

1. Acute respiratory event (primary outcome), *and*
2. Asthma-related outpatient department visit, *and*
3. Average daily dose of SABA >200 μg salbutamol/ >500 μg terbutaline

^f^GINA control status: poor asthma symptom control is defined as 3 out of 4 of the following:

1. “yes” to 3 RCP questions and/or
2. >2 puffs of SABA per week

Controlled = none of the questions have a “yes” response; Partly controlled = 1-2 of the questions have a “yes” response; Uncontrolled = 3-4 of the questions have a “yes” response

**S-Table 6A** Asthma-related outcomes in the period before and after MP-AzeFlu initiation among patients with allergic rhinitis and asthma who had a blood eosinophil count <0.25 10^9^/L before initiation with MP-AzeFlu (n=433)

| **Variable** | | **Pre-initiation MP-AzeFlu (N= 433)** | **Post-initiation MP-AzeFlu  (N= 433)** | **Change in outcomes** | **Effect^a^** | **P**^b^ |
| --- | --- | --- | --- | --- | --- | --- |
| Acute respiratory events, number | 0, n (%) | 211 (48.7) | 250 (57.7) | Stable: 214 (49.4%) | 11.8% | 0.0012 |
|  | 1, n (%) | 110 (25.4) | 97 (22.4) | Improved: 135 (31.2%) |  |  |
|  | 2, n (%) | 48 (11.1) | 41 (9.5) | Worsened: 84 (19.4%) |  |  |
|  | 3, n (%) | 41 (9.5) | 19 (4.4) |  |  |  |
|  | 4, n (%) | 6 (1.4) | 5 (1.2) |  |  |  |
|  | 5, n (%) | 11 (2.5) | 7 (1.6) |  |  |  |
|  | ≥6, n (%) | 6 (1.4) | 14 (3.2) |  |  |  |
| Asthma exacerbations based on ATS/ERS Force definition, number | 0, n (%) | 256 (59.1) | 285 (65.8) | Stable: 245 (56.6%) | 7.4% | 0.0283 |
|  | 1, n (%) | 98 (22.6) | 78 (18.0) | Improved: 110 (25.4%) |  |  |
|  | 2, n (%) | 38 (8.8) | 42 (9.7) | Worsened: 78 (18.0%) |  |  |
|  | 3, n (%) | 22 (5.1) | 8 (1.8) |  |  |  |
|  | 4, n (%) | 7 (1.6) | 2 (0.5) |  |  |  |
|  | 5, n (%) | 6 (1.4) | 6 (1.4) |  |  |  |
|  | ≥6, n (%) | 6 (1.4) | 12 (2.8) |  |  |  |
| Gina treatment step | 1, n (%) | 48 (11.1) | 73 (16.9) | Stable: 339 (78.3%) | 3.7% | 0.0906 |
|  | 2, n (%) | 83 (19.2) | 66 (15.2) | Improved: 55 (12.7%) |  |  |
|  | 3, n (%) | 54 (12.5) | 47 (10.9) | Worsened: 39 (9.0%) |  |  |
|  | 4, n (%) | 248 (57.3) | 245 (56.6) |  |  |  |
|  | 5, n (%) | 0 (0.0) | <5^c^ |  |  |  |
| Risk Domain Asthma Control^d^ | Controlled, n (%) | 204 (47.1) | 232 (53.6) | Stable: 313 (72.3%) | 6.5% | 0.0134 |
|  |  |  |  | Improved: 74 (17.1%) |  |  |
|  |  |  |  | Worsened: 46 (10.6%) |  |  |
| Overall Asthma Control^e^ | Controlled, n (%) | 128 (29.6) | 146 (33.7) | Stable: 325 (75.1%) | 4.1% | 0.0814 |
|  |  |  |  | Improved: 63 (14.5%) |  |  |
|  |  |  |  | Worsened: 45 (10.4%) |  |  |
| Average daily dose of SABA prescribed, salbutamol equivalent in µg/day | 0, n (%) | 54 (12.5) | 97 (22.4) | Stable: 195 (45.0%) | 12.0% | 0.0008 |
|  | 1-100, n (%) | 58 (13.4) | 44 (10.2) | Improved: 145 (33.5%) |  |  |
|  | 101-200, n (%) | 112 (25.9) | 79 (18.2) | Worsened: 93 (21.5) |  |  |
|  | 201-300, n (%) | 47 (10.9) | 64 (14.8) |  |  |  |
|  | 301-400, n (%) | 40 (9.2) | 44 (10.2) |  |  |  |
|  | >400, n (%) | 122 (28.2) | 105 (24.2) |  |  |  |
| >2 puffs of SABA per week | Yes, n (%) | 379 (87.5) | 336 (77.6) | Stable: 354 (81.8%) | 10.0% | <0.0001 |
|  |  |  |  | Improved: 61 (14.1%) |  |  |
|  |  |  |  | Worsened: 18 (4.1%) |  |  |
| Average daily dose of ICS | 0, n (%) | 139 (12.8) | 204 (18.8) | Stable: 278 (64.2%) | 2.6% | 0.4371 |
| based on collected | >0-≤250, n (%) | 531 (48.8) | 448 (41.2) | Improved: 83 (19.2%) |  |  |
| prescriptions, FP | >250-≤500, n (%) | 233 (21.4) | 258 (23.7) | Worsened: 72 (16.6%) |  |  |
| equivalent in µg/day | >500, n (%) | 185 (17.0) | 178 (16.4) |  |  |  |
| Gina level control^f^ | N (% non-missing) | 213 (49.2) | 213 (49.2) | Stable: 182 (85.4%) | 2.4% | 0.3692 |
|  | Controlled, n (%) | 16 (7.5) | 22 (10.3) | Improved: 18 (8.5%) |  |  |
|  | Partly controlled, n (%) | 192 (90.1) | 185 (86.9) | Worsened: 13 (6.1%) |  |  |
|  | Uncontrolled, n (%) | 5 (2.3) | 6 (2.8) |  |  |  |

Abbreviations: ATS/ERS: American Thoracic Society/European Respiratory Society; SABA: short-acting beta agonist

^a^Effect is expressed as % of patients that remained stable or improved from baseline to outcome 1-year periods.

^b^P-value for the Wilcoxon signed-ranks test (categorical variables), or the McNemar’s test (dichotomous variables), where appropriate

^c^Data suppressed to comply with privacy requirements (less than a count of 5 in a cell)

^d^Risk Domain asthma control (RDAC) (yes/no), defined as absence of any of the following events in the baseline year

1. Acute respiratory event (primary outcome as defined above), *and*
2. Asthma-related outpatient department visit

^e^Overall asthma control (OAC) (yes/no), defined as absence of any of the following events in the baseline year:

1. Acute respiratory event (primary outcome), *and*
2. Asthma-related outpatient department visit, *and*
3. Average daily dose of SABA >200 μg salbutamol/ >500 μg terbutaline

^f^GINA control status: poor asthma symptom control is defined as 3 out of 4 of the following:

1. “yes” to 3 RCP questions and/or
2. >2 puffs of SABA per week

Controlled = none of the questions have a “yes” response; Partly controlled = 1-2 of the questions have a “yes” response; Uncontrolled = 3-4 of the questions have a “yes” response

**S-Table 6B** Asthma-related outcomes in the period before and after MP-AzeFlu initiation among patients with allergic rhinitis and asthma who had a blood eosinophil count >0.25 10^9^/L before initiation with MP-AzeFlu (n=488)

| **Variable** | | **Pre-initiation MP-AzeFlu (N= 488)** | **Post-initiation MP-AzeFlu (N= 488)** | **Change in outcomes** | **Effect^a^** | **P**^b^ |
| --- | --- | --- | --- | --- | --- | --- |
| Acute respiratory events, number | 0, n (%) | 251 (51.4) | 270 (55.3) | Stable: 242 (49.6%) | 3.6% | 0.4758 |
|  | 1, n (%) | 109 (22.3) | 95 (19.5) | Improved: 132 (27.0%) |  |  |
|  | 2, n (%) | 63 (12.9) | 52 (10.7) | Worsened: 114 (23.4%) |  |  |
|  | 3, n (%) | 23 (4.7) | 26 (5.3) |  |  |  |
|  | 4, n (%) | 14 (2.9) | 18 (3.7) |  |  |  |
|  | 5, n (%) | 15 (3.1) | 8 (1.6) |  |  |  |
|  | ≥6, n (%) | 13 (2.7) | 19 (3.9) |  |  |  |
| Asthma exacerbations based on ATS/ERS Force definition, number | 0, n (%) | 292 (59.8) | 300 (61.5) | Stable: 272 (55.8%) | 0.0% | 0.7133 |
|  | 1, n (%) | 102 (20.9) | 86 (17.6) | Improved: 108 (22.1%) |  |  |
|  | 2, n (%) | 45 (9.2) | 46 (9.4) | Worsened: 108 (22.1%) |  |  |
|  | 3, n (%) | 15 (3.1) | 18 (3.7) |  |  |  |
|  | 4, n (%) | 17 (3.5) | 15 (3.1) |  |  |  |
|  | 5, n (%) | 8 (1.6) | 7 (1.4) |  |  |  |
|  | ≥6, n (%) | 9 (1.9) | 16 (3.3) |  |  |  |
| Gina treatment step | 1, n (%) | 45 (9.2) | 74 (15.2) | Stable: 384 (78.7%) | 5.3% | 0.0150 |
|  | 2, n (%) | 111 (22.7) | 87 (17.8) | Improved: 65 (13.3%) |  |  |
|  | 3, n (%) | 63 (12.9) | 55 (11.3) | Worsened: 39 (8.0%) |  |  |
|  | 4, n (%) | 269 (55.1) | 270 (55.3) |  |  |  |
|  | 5, n (%) | 0 (0.0) | <5^c^ |  |  |  |
| Change in Gina treatment step | Stable, n (%) | - | 384 (78.7) | - | - |  |
|  | Step-down, n (%) | - | 65 (13.3) |  |  |  |
|  | Step-up, n (%) | - | 39 (8.0) |  |  |  |
| Risk Domain Asthma Control^d^ | Controlled, n (%) | 239 (49.0) | 261 (53.5) | Stable: 342 (70.1%) | 4.5% | 0.0686 |
|  |  |  |  | Improved: 84 (17.2%) |  |  |
|  |  |  |  | Worsened: 62 (12.7%) |  |  |
| Overall Asthma Control^e^ | Controlled, n (%) | 126 (25.8) | 145 (29.7) | Stable: 381 (78.1%) | -3.9% | 0.0814 |
|  |  |  |  | Improved: 44 (9.0%) |  |  |
|  |  |  |  | Worsened: 63 (12.9%) |  |  |
| Average daily dose of SABA prescribed, salbutamol equivalent in µg/day | N (% non-missing) | 45 (9.2) | 45 (9.2) | Stable: 34 (75.6%) | - | 0.5488 |
|  | 0, n (%) | 52 (10.7) | 85 (17.4) | Stable: 212 (43.4%) | 10.2% | 0.0023 |
|  | 1-100, n (%) | 62 (12.7) | 51 (10.5) | Improved: 163 (33.4%) |  |  |
|  | 101-200, n (%) | 101 (20.7) | 84 (17.2) | Worsened: 113 (23.2) |  |  |
|  | 201-300, n (%) | 66 (13.5) | 75 (15.4) |  |  |  |
|  | 301-400, n (%) | 49 (10.0) | 55 (11.3) |  |  |  |
|  | >400, n (%) | 158 (32.4) | 138 (28.3) |  |  |  |
| >2 puffs of SABA per week | Yes, n (%) | 436 (89.3) | 403 (82.6) | Stable: 405 (83.0%) | 6.8% | 0.0004 |
|  |  |  |  | Improved: 58 (11.9%) |  |  |
|  |  |  |  | Worsened: 25 (5.1%) |  |  |
| Average daily dose of ICS  based on collected  prescriptions, FP  equivalent in µg/day | 0, n (%) | 50 (10.2) | 82 (16.8) | Stable: 299 (61.3%) | 6.3% | 0.0028 |
|  | >0-≤250, n (%) | 220 (45.1) | 190 (38.9) | Improved: 110 (22.5%) |  |  |
|  | >250-≤500, n (%) | 125 (25.6) | 119 (24.4) | Worsened: 79 (16.2%) |  |  |
|  | >500, n (%) | 93 (19.1) | 97 (19.9) |  |  |  |
| Gina level control^f^ | N (% non-missing) | 260 (53.3) | 260 (53.3) | Stable: 202 (77.7%) | -1.5% | 0.5994 |
|  | Controlled, n (%) | 26 (10.0) | 26 (10.0) | Improved: 27 (10.4%) |  |  |
|  | Partly controlled, n (%) | 226 (86.9) | 222 (85.4) | Worsened: 31 (11.9%) |  |  |
|  | Uncontrolled, n (%) | 8 (3.1) | 12 (4.6) |  |  |  |

Abbreviations: ATS/ERS: American Thoracic Society/European Respiratory Society; SABA: short-acting beta agonist

^a^Effect is expressed as % of patients that remained stable or improved from baseline to outcome 1-year periods.

^b^P-value for the Wilcoxon signed-ranks test (categorical variables), or the McNemar’s test (dichotomous variables), where appropriate

^c^Data suppressed to comply with privacy requirements (less than a count of 5 in a cell)

^d^Risk Domain asthma control (RDAC) (yes/no), defined as absence of any of the following events in the baseline year

1. Acute respiratory event (primary outcome as defined above), *and*
2. Asthma-related outpatient department visit

^e^Overall asthma control (OAC) (yes/no), defined as absence of any of the following events in the baseline year:

1. Acute respiratory event (primary outcome), *and*
2. Asthma-related outpatient department visit, *and*
3. Average daily dose of SABA >200 μg salbutamol/ >500 μg terbutaline

^f^GINA control status: poor asthma symptom control is defined as 3 out of 4 of the following:

1. “yes” to 3 RCP questions and/or
2. >2 puffs of SABA per week

Controlled = none of the questions have a “yes” response; Partly controlled = 1-2 of the questions have a “yes” response; Uncontrolled = 3-4 of the questions have a “yes” response

1. Asthma-Related Hospitalisations: consists of a definite Asthma Hospital Admission; OR a generic hospitalisation Read code which has been recorded on the same day as a Lower Respiratory Consultation. [↑](#footnote-ref-1)
2. Asthma-Related Accident and Emergency attendances: consists of a definite Asthma Emergency Attendance; OR a generic emergency hospital Read code which has been recorded on the same day as a Lower Respiratory Consultation. [↑](#footnote-ref-2)
3. Asthma-Related Hospitalisations: consists of a definite Asthma Hospital Admission; OR a generic hospitalisation Read code which has been recorded on the same day as a Lower Respiratory Consultation. [↑](#footnote-ref-3)
4. Asthma-Related Accident and Emergency attendances: consists of a definite Asthma Emergency Attendance; OR a generic emergency hospital Read code which has been recorded on the same day as a Lower Respiratory Consultation. [↑](#footnote-ref-4)
